# A Network of microRNAs Acts to Promote Cell Cycle Exit and Differentiation of Human Pancreatic Endocrine Cells

**DOI:** 10.1016/j.isci.2019.10.063

**Published:** 2019-11-01

**Authors:** Wen Jin, Francesca Mulas, Bjoern Gaertner, Yinghui Sui, Jinzhao Wang, Ileana Matta, Chun Zeng, Nicholas Vinckier, Allen Wang, Kim-Vy Nguyen-Ngoc, Joshua Chiou, Klaus H. Kaestner, Kelly A. Frazer, Andrea C. Carrano, Hung-Ping Shih, Maike Sander

**Affiliations:** 1 Departments of Pediatrics and Cellular & Molecular Medicine, Pediatric Diabetes Research Center, University of California, San Diego, La Jolla, CA 92093, USA; 2 Department of Genetics and Institute for Diabetes, Obesity, and Metabolism, University of Pennsylvania, Perelman School of Medicine, Philadelphia, PA 19104, USA; 3 Department of Pediatrics, Institute for Genomic Medicine, University of California, San Diego, La Jolla, CA 92093, USA; 4 Department of Translational Research and Cellular Therapeutics, Diabetes and Metabolic Research Institute, Beckman Research Institute, City of Hope, Duarte, CA 91010, USA

**Keywords:** Molecular Mechanism of Gene Regulation, Molecular Network, Endocrinology, Stem Cells Research

## Abstract

Pancreatic endocrine cell differentiation is orchestrated by the action of transcription factors that operate in a gene regulatory network to activate endocrine lineage genes and repress lineage-inappropriate genes. MicroRNAs (miRNAs) are important modulators of gene expression, yet their role in endocrine cell differentiation has not been systematically explored. Here we characterize miRNA-regulatory networks active in human endocrine cell differentiation by combining small RNA sequencing, miRNA over-expression, and network modeling approaches. Our analysis identified Let-7g, Let-7a, miR-200a, miR-127, and miR-375 as endocrine-enriched miRNAs that drive endocrine cell differentiation-associated gene expression changes. These miRNAs are predicted to target different transcription factors, which converge on genes involved in cell cycle regulation. When expressed in human embryonic stem cell-derived pancreatic progenitors, these miRNAs induce cell cycle exit and promote endocrine cell differentiation. Our study delineates the role of miRNAs in human endocrine cell differentiation and identifies miRNAs that could facilitate endocrine cell reprogramming.

## Highlights

• Genome-wide identification of miRNAs regulated in human endocrine cell development
• Endocrine-enriched miRNAs promote cell cycle exit and endocrine cell formation
• Network modeling predicts miRNA-regulated TFs and downstream cell cycle regulators

## Graphical Abstract

**Figure:**
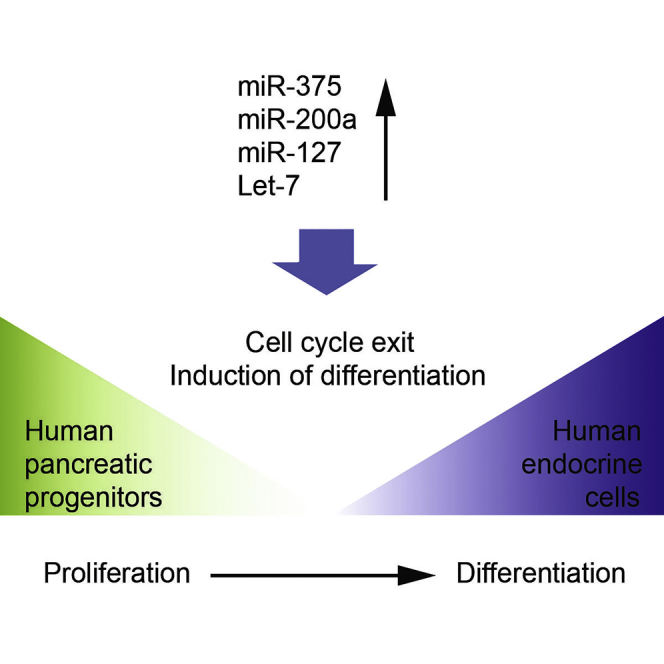

## Introduction

The potential to generate pancreatic beta cells from human pluripotent stem cells (hPSCs) or via cell reprogramming from other cell sources holds promise for modeling causes of diabetes and cell replacement therapies (Benthuysen et al., 2016). Knowledge of the molecular underpinnings of pancreas and beta cell development has enabled some success in developing beta cell reprogramming and directed differentiation strategies. In particular, the identification of transcription factors (TFs) governing cell fate decisions has been instrumental for cell reprogramming approaches (Benthuysen et al., 2016). Although TFs play a major role in orchestrating gene expression changes during developmental transitions, recent evidence also shows significant roles for other regulators such as small RNAs.

MicroRNAs (miRNAs) are a group of small non-coding RNAs (∼22 nucleotides) with known roles in the regulation of gene expression in development, mature cell function, and disease (Vidigal and Ventura, 2015). Studies in mice and zebrafish have demonstrated important roles for miRNAs in pancreatic endocrine cell development and beta cell function (Kaspi et al., 2014). Pancreatic progenitor cell-specific deletion of *Dicer1,* an enzyme that is universally required for the functional maturation of miRNAs, results in reduced endocrine cell numbers (Lynn et al., 2007), whereas *Dicer1* disruption in beta cells impairs insulin biogenesis (Melkman-Zehavi et al., 2011). At the level of individual miRNAs, miR-375 (Kloosterman et al., 2007, Poy et al., 2009) and miR-7 (Kredo-Russo et al., 2012, Latreille et al., 2014) have been identified as regulators of beta cell differentiation and function.

Generally, miRNAs are thought to repress target mRNAs and act by destabilizing mRNAs through base pairing between the miRNA seed sequence (nucleotides at position 2–8) and a complementary sequence in the target mRNA (Guo et al., 2010, Lim et al., 2005). However, recent evidence suggests that miRNAs can also activate gene expression (Jopling et al., 2008, Valinezhad Orang et al., 2014, Vasudevan et al., 2007). The effects of individual miRNAs on gene expression are generally small, which has led to the concept that miRNAs fine-tune gene expression rather than acting as genetic switches (Vidigal and Ventura, 2015). Consistent with this idea, miRNAs have been shown to promote cell differentiation and to facilitate cell reprogramming when force expressed in conjunction with lineage-determining TFs (Chen et al., 2004, Chen et al., 2006, Dey et al., 2012, Lim et al., 2005, Nam et al., 2013, Yoo et al., 2011). Mechanistically, each miRNA has the ability to repress hundreds of mRNA targets, and multiple miRNAs often converge on a single pathway to promote a common developmental outcome (Lim et al., 2005, Vidigal and Ventura, 2015). Therefore, a comprehensive understanding of context-specific contributions of miRNAs to gene regulation requires a systems-level approach where all miRNAs and their targets are considered.

In this study we used genome-wide small RNA sequencing to identify candidate miRNAs with possible roles in human endocrine cell differentiation. By comparing miRNA profiles of hPSC-derived pancreatic progenitors and human cadaveric beta and alpha cells genome-wide, we identified miRNAs that are induced during endocrine cell differentiation. Through gain-of-function experiments during hPSC differentiation, we show that islet cell-enriched miRNAs act to promote cell cycle exit and hence islet cell differentiation. Integrating RNA-seq, CLIP-seq, and chromatin state data, we applied a network modeling approach to identify candidate miRNA-regulated TFs that explain the impact of islet cell-enriched miRNAs on cell cycle regulation during endocrine cell differentiation. Our findings provide a systems-level view of how miRNAs regulate human endocrine cell differentiation, which has implications for programming islet endocrine cells from hPSCs or other cell sources.

## Results

### Identification of miRNAs Up-Regulated during Endocrine Cell Differentiation

**Figure 1:**
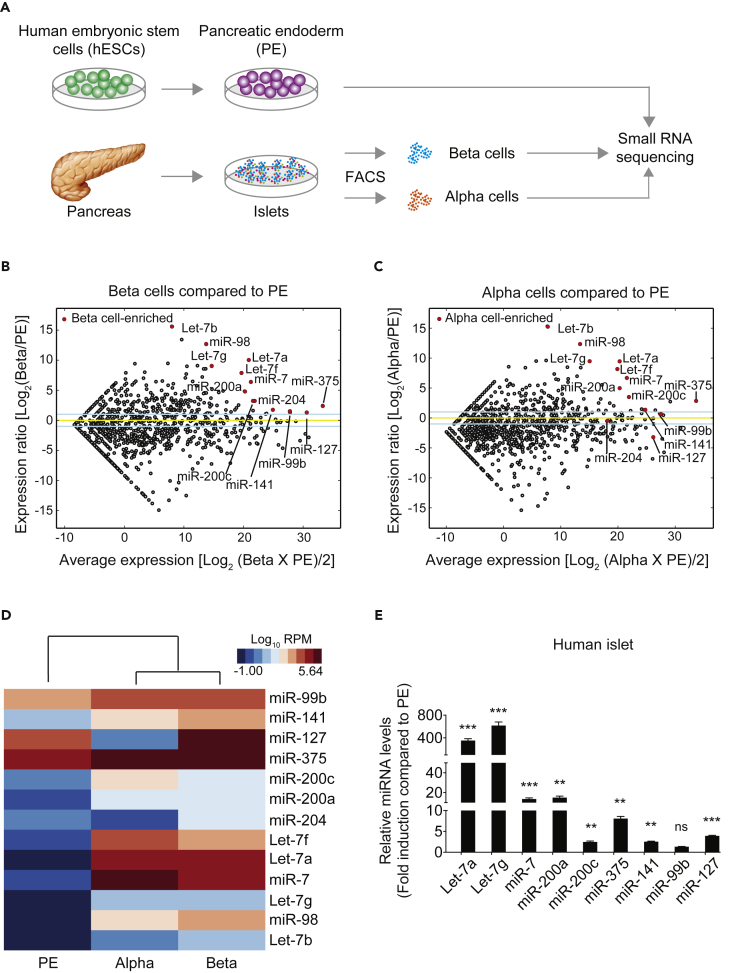
Identification of miRNAs Up-Regulated during Endocrine Cell Differentiation of Human Pancreatic Progenitor Cells (A) Workflow for genome-wide small RNA profiling of pancreatic progenitors (pancreatic endoderm, PE) and endocrine islet cells. PE cells were differentiated from human embryonic stem cells (hESCs), and human alpha and beta cells were isolated from cadaveric human islets by fluorescence-activated cell sorting (FACS). (B and C) MA plots comparing miRNA expression levels in PE cells and beta cells (B) or PE cells and alpha cells (C). miRNAs with higher expression in beta and alpha cells than PE are indicated by red circles in B and C, respectively. Blue lines indicate 2-fold change in miRNA expression; yellow line indicates no change. (D) Heatmap comparing expression levels in PE, alpha cells, and beta cells of the thirteen most highly enriched miRNAs in beta cells compared with PE cells. (E) Relative expression of indicated miRNAs determined by Taqman qPCR in PE cells and human islets. Data are shown as mean ± S.E.M. (n = 3 biological replicates). ns, not significant; **p < 0.01, ***p < 0.001; Student's t test. See also Figure S1 and Table S1.

To identify miRNAs that are regulated during pancreatic beta cell differentiation, we conducted genome-wide small RNA sequencing in pancreatic progenitor cells derived from CyT49 human embryonic stem cells (hESCs) (Figure S1) and primary beta cells isolated from cadaveric human islets by fluorescence-activated cell sorting (Kameswaran et al., 2014) (Figure 1A). Although both up- and down-regulated miRNAs could have roles in beta cell differentiation, we here focused on miRNAs that increase in expression during endocrine cell differentiation. By comparing expression levels of individual miRNAs in beta cells and pancreatic endoderm stage (PE) cells, we defined miRNAs induced during beta cell differentiation. This analysis revealed 14 miRNAs that were more highly expressed in beta cells than in PE cells (>5,000 sequence reads in beta cells; > 2.3-fold increase; Figure 1B and Tables S1A and S1B). With the exception of miR-127, miR-204, and miR-99b, the same miRNAs also exhibited higher expression in sorted alpha cells compared with PE cells (Figure 1C and Tables S1A and S1C), suggesting shared roles for most miRNAs in the development of both endocrine cell types. Among the miRNAs induced during endocrine cell differentiation were miR-375, miR-200a/c, and miR-7, which have reported roles in beta cell development, proliferation, function, and survival in mice (Belgardt et al., 2015, Kloosterman et al., 2007, Kredo-Russo et al., 2012, Latreille et al., 2014, Nieto et al., 2012, Poy et al., 2004, Poy et al., 2009, Wang et al., 2013). Most notable was the significantly higher expression of members of the Let-7 miRNA family in both beta and alpha cells compared with PE cells, including Let-7a, Let-7b, Let-7f, Let-7g, and miR-98 (Figures 1B and 1D and Tables S1A and S1B). We confirmed the results from the small RNA sequencing by comparing miRNA levels in PE cells and human cadaveric islets using the Taqman miRNA assay (Figure 1E).

### Identifying miRNAs Regulating Human Endocrine Cell Differentiation

To identify mRNAs regulated by these miRNAs, we selected several miRNAs for over-expression in hESC-derived PE cells. We included the top three beta cell-enriched miRNAs (miR-375, miR-127, and Let-7a), as well as Let-7g and miR-200a, as they are highly induced during endocrine cell differentiation. miR-7 was not included because of its inhibitory role in endocrine cell differentiation in mice (Kredo-Russo et al., 2012).

**Figure 2:**
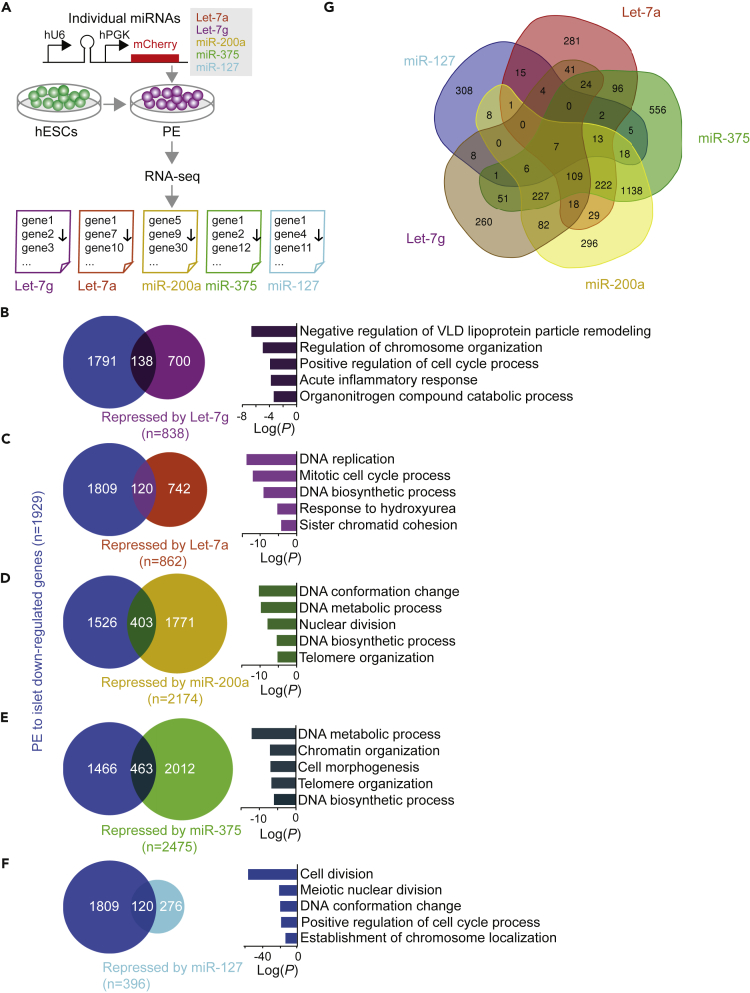
Endocrine Cell-Enriched miRNAs Regulate Expression of Cell Cycle Genes in Pancreatic Progenitor Cells (A) Workflow to identify genes repressed by each indicated miRNA after lentiviral transduction of hESC-derived pancreatic endoderm (PE) cells. Transduced cells were sorted based on mCherry after 48 h, RNA-seq analysis performed (n = 3 biological replicates), and down-regulated genes identified. (B–F) Venn diagrams showing the overlap between genes down-regulated in islets (n = 3) compared with PE (n = 2) (blue) and genes repressed by Let-7g (purple, B), Let-7a (red, C), miR-200a (yellow, D), miR-375 (green, E), or miR-127 (light blue, F). Top five GO categories enriched among genes repressed by the miRNA and down-regulated in islets compared with PE are shown on the right. (G) Venn diagram showing overlap between miRNA-repressed genes. See also Figure S2, Tables S2, and S3.

To determine the effects of these miRNAs on gene expression, we next over-expressed Let-7g, Let-7a, miR-200a, miR-375, and miR-127 individually in hESC-derived PE cells (Figure 2A). For these studies, we chose PE cells derived from H1 hESCs because a recently published protocol showed very efficient differentiation of H1 hESCs into beta-like cells *in vitro* (Rezania et al., 2014). Since our genome-wide small RNA sequencing was performed in PE cells from CyT49 hESCs (Figure 1), we first confirmed that H1 and CyT49 hESC-derived PE cells have similar molecular features. Similar to CyT49 hESC-derived PE cells (Figure S1), 98% of H1 hESC-derived PE cells expressed the pancreatic progenitor marker PDX1 (Figures S2A and S2B). In addition, RNA-seq analysis showed highly concordant transcriptome profiles of H1 and CyT49 hESC-derived PE cells [(R) > 0.92; Figure S2C]. Furthermore, we confirmed that Let-7g, Let-7a, miR-200a, and miR-375 were expressed at similarly low levels in H1 and CyT49 hESC-derived PE cells (Figure S2D).

Inclusion of an mCherry reporter into the miRNA constructs allowed us to monitor transduction efficiencies in PE stage cultures and to isolate transduced cells by FACS. We observed 13%–20% mCherry^+^ PE cells 2 days after transduction, and this number increased to 34%–49% 6 days after transduction (Figure S2E). The increase is likely explained by the lentiviral expression vector requiring more than 2 days to reach maximum expression. To identify miRNA targets, we analyzed sorted mCherry^+^ PE cells 2 days after transduction, reasoning that this early time point is best suited for studying the direct effects of miRNA on gene expression. As expected, Let-7g, Let-7a, miR-200a, miR-375, and miR-127 were each expressed at significantly higher levels in cells transduced with the miRNA-expressing vector compared with control vector-transduced cells (Figure S2F). Furthermore, forced expression of Let-7g, Let-7a, miR-200a, miR-375, or miR-127 in hESC-derived PE repressed the expression of genes (p < 0.05, permutation test, Tables S2A–S2E) that were down-regulated between PE and islets (Figure S2G), suggesting that these miRNAs could contribute to gene expression changes during islet cell differentiation.

### Islet Cell-Enriched miRNAs Regulate Expression of Cell Cycle Genes in Pancreatic Progenitor Cells

To identify miRNA-regulated transcripts with likely roles in endocrine cell differentiation, we analyzed sets of genes that were down-regulated by forced expression of each miRNA (p < 0.05, permutation test) and also down-regulated in islets as compared with PE cells (p < 0.05, permutation test, Figures 2B–2F). These mRNA subsets comprised 16.5% of Let-7g-, 13.9% of Let-7a-, 18.5% of miR-200a-, 18.7% of miR-375-, and 30.3% of miR-127-repressed mRNAs in PE cells. We then performed Gene Ontology (GO) analysis to define the biological processes regulated by mRNAs that are repressed by individual miRNAs and are also expressed at lower level in islet than PE cells. The top five enriched GO categories for each one of these miRNA-regulated sets of mRNAs comprised processes associated with DNA replication and regulation of the cell cycle (Figures 2B–2F and Tables S3A–S3E). Given that endocrine cell formation is associated with cell cycle exit (Kim et al., 2015, Miyatsuka et al., 2011, Piccand et al., 2014), these findings suggest that miRNAs could control endocrine cell differentiation by regulating mRNAs involved in cell cycle control. The finding that all five miRNAs regulate cell cycle-associated transcripts raised the question of whether they share similar target genes. Analysis of the extent of overlap between the mRNAs down-regulated by Let-7g, Let-7a, miR-200a, miR-375, and miR-127 revealed a modest number of shared targets (Figure 2G). Only seven mRNAs (*ZNF239, PIF1, CDC45, TMEM114, HIST1H4H, MRPS25,* and *ESPL1*) were repressed by all five miRNAs, indicating distinct regulatory roles for each one of the miRNAs. Together, these results suggest distinct but converging miRNA targets in regulating cell division in pancreatic progenitors.

**Figure 3:**
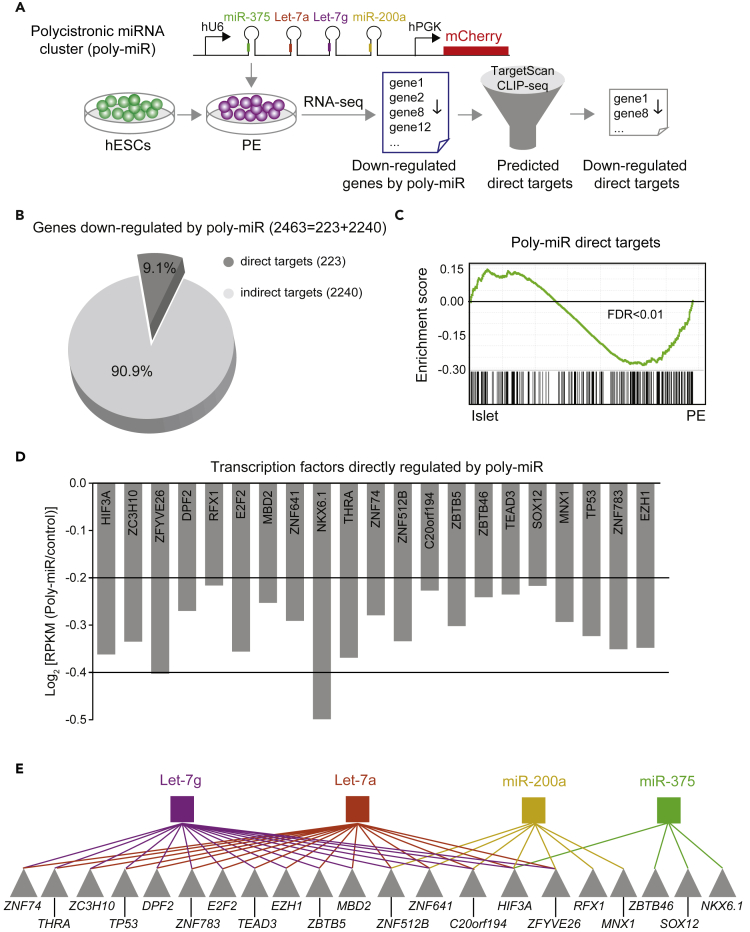
Identification of Putative Direct miRNA Target mRNAs in Pancreatic Progenitor Cells (A) Workflow to identify repressed genes after transduction of hESC-derived pancreatic endoderm (PE) cells with a lentivirus expressing a polycistronic construct for the indicated miRNAs (poly-miR) and mCherry. Transduced cells were sorted after 48 h, RNA-seq analysis performed (n = 3 biological replicates), and down-regulated genes identified. Direct targets of candidate miRNAs were identified based on TargetScan and CLIP-seq analysis. (B) Pie graph showing percentage of direct (dark gray) and indirect (light gray) targets of candidate miRNAs repressed by poly-miR construct. (C) GSEA plot showing enrichment of 223 direct target genes of Let-7g, Let-7a, miR-200a, and miR-375 in islets (n = 3) compared with PE (n = 2). False Discovery Rate (FDR) is shown. (D) mRNA expression levels of transcription factors directly targeted by Let-7g, Let-7a, miR-200a, and miR-375 measured in reads per kilobase per million reads mapped (RPKM). (E) Predicted network of transcription factors downstream of miRNAs. Transcription factors are indicated by gray triangles, and individual miRNAs are indicated by colored squares. See also Figure S3, Tables S2, S3, and S4.

Since all candidate miRNAs appeared to regulate different aspects of cell cycle progression, we sought to gain further insight into how input from the different miRNAs converges on cell cycle regulation. To study the combined effect of miRNAs, we generated a “polycistronic” miRNA (poly-miR) lentiviral construct that drives the expression of Let-7g, Let-7a, miR-200a, and miR-375 under the control of a single promoter. miR-127 was excluded because overall it repressed fewer genes than the other miRNAs (Figure 2F). We expressed the poly-miR construct in H1 hESC-derived PE cells and analyzed the transcriptome two days after transduction (Figure 3A). miRNA expression analysis in mCherry-sorted cells revealed that Let-7g, Let-7a, miR-200a, and miR-375 were each significantly higher expressed in poly-miR- than vector-only-transduced PE cells (Figures S3A and S3B). Expression of the poly-miR construct in PE cells resulted in down-regulation of 2,463 transcripts (p < 0.05; permutation test). Consistent with the results from expression of individual miRNAs (Figure S2G), poly-miR-repressed mRNAs (p < 0.05, permutation test, Table S2F) were highly enriched for mRNAs with higher expression in PE compared with islets (Figure S3C and Table S2G). Of the 2,463 poly-miR-repressed mRNAs, 388 were also down-regulated during the transition of PE to islet (Figure S3D). As predicted, genes involved in cell cycle processes were overrepresented among these 388 mRNAs (Figure S3D and Table S3F).

### Endocrine-Enriched miRNAs Regulate Cell Cycle-Associated Transcription Factors

To decipher mechanisms by which Let-7g, Let-7a, miR-200a, and miR-375 regulate cell cycle genes, we sought to distinguish direct and indirect targets of the four miRNAs (Figure 3A). We defined putative direct targets as poly-miR-repressed mRNAs (p < 0.05, permutation test, Table S2F) predicted to be direct targets by TargetScan (based on matching sequence to the miRNA seed region) and/or exhibiting binding to the RNA-binding protein Argonaute, as determined by CLIP-seq in human islets (Kameswaran et al., 2014). From this analysis, 223 putative direct target mRNAs were identified (Figure 3B and Table S4A). Of these, all were predicted by TargetScan and 35 also by HITS-CLIP. These 223 genes represent 9.1% of all poly-miR-repressed genes. Reinforcing the potential relevance of these predicted direct miRNA targets for endocrine cell development, GSEA analysis showed significantly lower expression of these genes in islets than in PE cells (Figure 3C).

To determine whether miRNAs are direct regulators of cell cycle-associated mRNAs in PE cells, we analyzed enriched GO terms among the 223 predicted direct miRNA targets. We found no enrichment of categories linked to cell cycle-related processes (Table S4B). Moreover, many of the cell cycle regulators that were repressed by the poly-miR, including *CCND3*, *CDC45*, *MCM7*, and *CKS1B* (Table S2F), were not among the predicted direct miRNA targets (Table S4A). Thus, cell cycle-associated transcripts appear to be indirectly regulated by the miRNAs. We postulated that this indirect effect of miRNAs on the expression of cell cycle genes could be mediated through the regulation of TFs. Consistent with this hypothesis, 21 TFs were among the 223 putative direct miRNA targets (Figure 3D and Tables S4A and S4C). A striking finding was that many of these TFs have documented roles in cell cycle regulation, including *E2F2*, which is part of the complex controlling cell cycle progression, and numerous TFs are known to regulate cell growth (e.g., *ZC3H10*, *ZNF783*, *ZBTB46*, *ZBTB5*, *ZFYVE26*, *TP53, EZH1, HIF3A, DPF2, TEAD3*). In addition, TFs predicted to be directly regulated by the miRNAs included TFs involved in the regulation of endocrine cell development and maturation, such as *NKX6.1* (Schaffer et al., 2013, Taylor et al., 2013) and the thyroid hormone receptor *THRA*, consistent with the role of thyroid hormone in beta cell maturation (Matsuda et al., 2017). Reflective of their shared seed sequence, TFs that are predicted to be directly regulated by Let-7g and Let-7a showed complete overlap, whereas miR-200a and miR-375 mostly regulated separate sets of TFs (Figure 3E). This analysis indicates that Let-7g, Let-7a, miR-200a, and miR-375 might jointly change the transcriptional landscape in PE cells by down-regulating expression of different sets of TFs.

### miRNAs Regulate a Network of Cell Cycle Genes in Pancreatic Progenitor Cells

**Figure 4:**
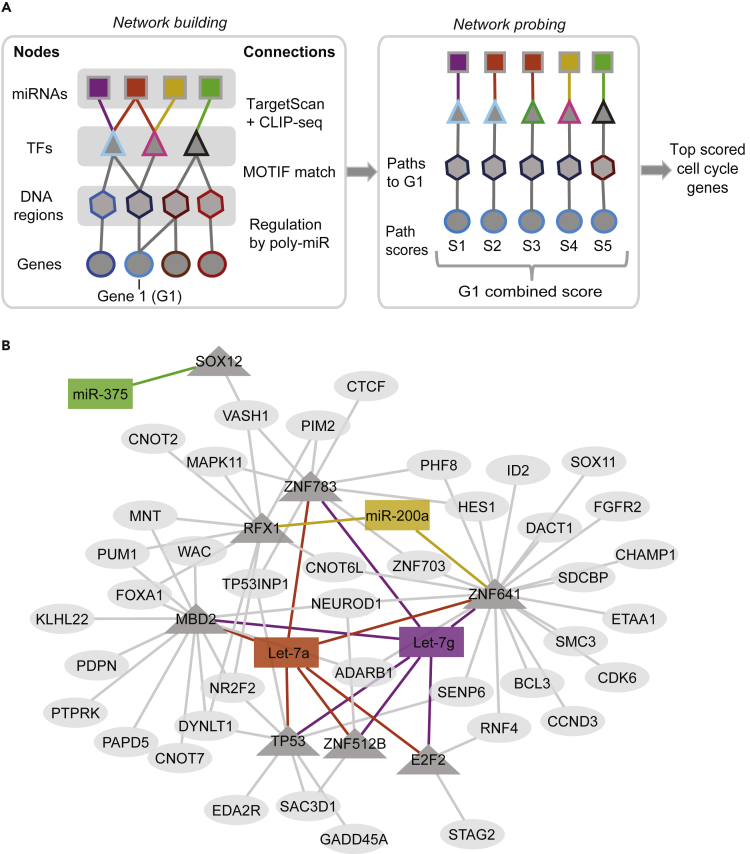
Endocrine Cell-Enriched miRNAs Regulate a Network of Cell Cycle Genes (A) Schematic of approach to identify core network of miRNA-regulated transcription factors and down-stream target genes. Building of network (left) and probing of network (right) is summarized. The nodes of the graph represent miRNAs (squares; Let-7g [purple], Let-7a [red], miR-200a [yellow], and miR-375 [green]), TFs (triangles), TF-binding regions (hexagons), and genes (circles). (B) Predicted network of 40 highest scoring cell cycle genes based on network in (A) with miRNAs depicted as rectangles, TFs as triangles, and genes as ovals. TF, transcription factor; G, gene; S, score. See also Figure S4, Tables S5, and S6.

Having identified a set of TFs as potential direct miRNA targets, we next sought to determine whether these TFs could act downstream of the miRNAs to regulate cell cycle genes. To test this, we constructed and subsequently probed a miRNA-gene regulatory network, linking the four candidate miRNAs and their putative direct TF targets to poly-miR-regulated genes predicted to be target genes of the TFs (Figures 4A, S4A, and S4B). First, to identify TF-binding events close to poly-miR-regulated genes (down- and up-regulated), we used ATAC-seq data from PE cells and islets and mapped open chromatin regions surrounding transcriptional start sites (TSSs; closest within 10 kb) of these genes (n = 241,922 sites; FDR <0.01, MACS2) (Figures 4A and S4A; see Transparent Methods). Second, to pinpoint identified candidate TF-bound regions with likely impact on gene regulation during the PE to islet transition, we identified ATAC sites exhibiting dynamics in histone modifications between PE cells and islets. We focused on H3K4me3 and H3K27ac, two highly dynamic histone modifications during development (Wang et al., 2015, Xie et al., 2013) that have been associated with active promoters (H3K4me3) and active promoters and enhancers (H3K27ac) (Creyghton et al., 2010, Heintzman et al., 2009). We then tested whether changes in these histone marks are accompanied by expression changes of proximal genes. As predicted, an increase in H3K4me3 and H3K27ac deposition in PE compared with islets was associated with higher mRNA levels (p = 5.3 × 10^−134^; Mann-Whitney test), whereas a decrease was associated with lower mRNA levels (p = 1.9 × 10^−36^). Finally, to construct the network, we linked open chromatin regions with dynamic histone marks to miRNA-regulated TFs by identifying those regions with a matching TF-binding motif. Validating our miRNA-gene regulatory network, GO analysis showed that the 1,307 genes comprising the network were enriched for cell cycle regulators (Figure S4C and Table S5).

Having validated our approach of linking putative TF binding events to changes in gene transcription during the PE to islet transition, we next assembled all data into a structured graph (Figures 4A and S4A) consisting of different types of nodes that represent the individual datasets, namely, the four candidate miRNAs (squares, Let-7g, Let-7a, miR-200a, and miR-375), their predicted target TFs (triangles), predicted TF-binding regions (hexagons), and indirect miRNA target genes (circles). Each connection between nodes (i.e., edge) was given a score representing the strength of their association, as inferred from miRNA-target databases (a), from algorithms matching TF motifs to DNA sequences (b), or from differential regulation of the connected gene (c) (see Figure S4A for details). A combined score was then computed for each possible path in the network from a miRNA to a gene. The score for an individual gene (G1) regulated by a miRNA is the sum of the edge scores (S1 = a1 + b1 + c1). To account for effects of more than one miRNA on an individual gene, we then computed a combined score representing the connectivity of the four miRNAs to G1. According to this scoring system, a higher rank is assigned to genes with strong connectivity of individual miRNA-mediated paths and characterized by an effect of more than one miRNA (Figure S4B and Table S6). The resulting network of the 40 highest scoring cell cycle genes demonstrates direct connectivity of the four miRNAs with cell cycle regulators through several TFs, including *SOX12, RFX1, TP53, E2F2, MBD2, ZNF512B, ZNF783,* and *ZNF641* (Figure 4B). Of interest is the identification of *NEUROD1* as an indirect miRNA target of Let-7 miRNAs and miR200a. Neurod1 is a TF that has been shown to induce cell cycle exit and to regulate endocrine cell differentiation in model organisms (Ahnfelt-Ronne et al., 2007, Mutoh et al., 1998). Our network analysis identifies a core network of miRNAs, TFs as their putative direct targets, and down-stream genes with likely roles in cell cycle regulation and endocrine cell differentiation.

### miRNAs Regulate Endocrine Cell Differentiation by Promoting Cell Cycle Exit

**Figure 5:**
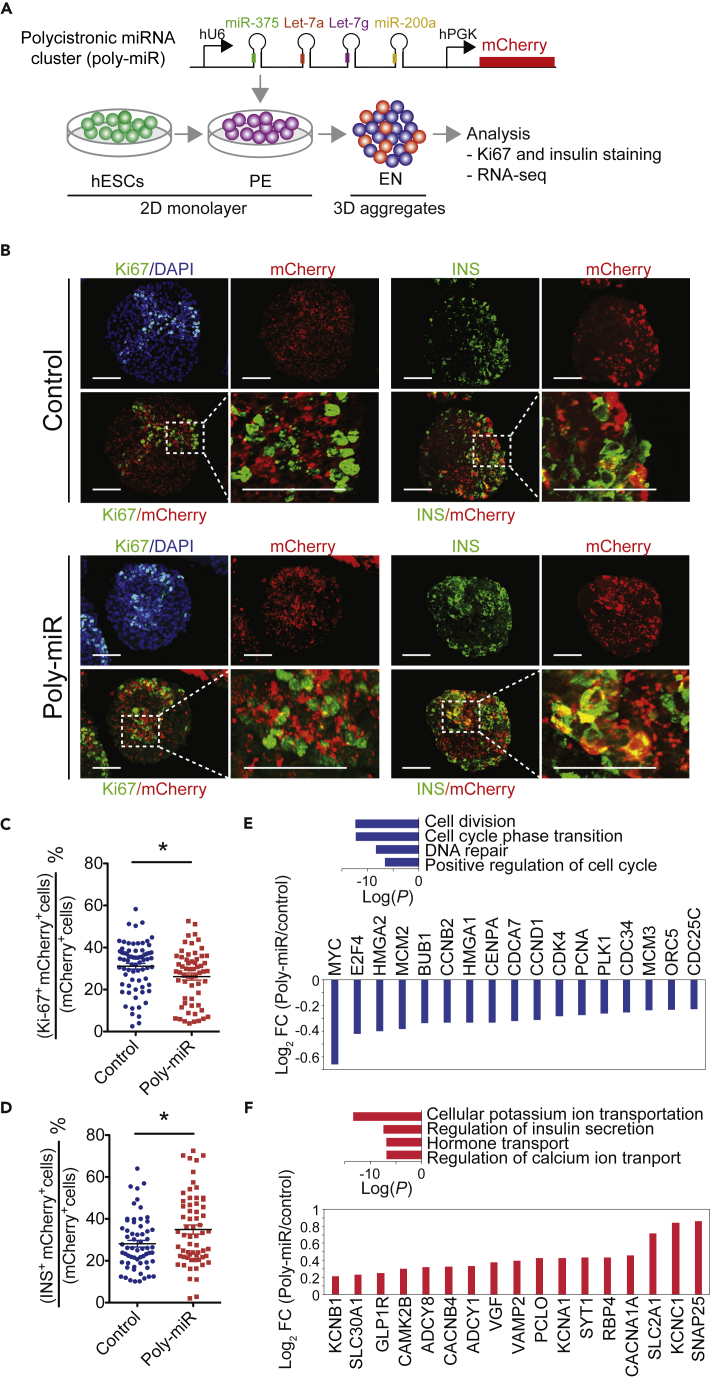
Endocrine Cell-Enriched miRNAs Regulate Cell Cycle Exit and Endocrine Cell Differentiation (A) Workflow to test effects of miRNAs on cell proliferation and endocrine cell differentiation during the transition of hESC-derived pancreatic endoderm (PE) to the early endocrine (EN) cell stage. Early PE stage cells were transduced with a lentivirus expressing a polycistronic construct for the indicated miRNAs (poly-miR) and mCherry, cultured in 2D until the end of the PE stage, aggregated, differentiated in 3D to the EN stage, sectioned, and stained for Ki-67 and insulin (INS). (B) Representative images showing immunofluorescence staining for Ki-67 (left) and INS (right) together with mCherry and DAPI at the EN stage for control vector (top) or poly-miR (bottom) transduced aggregates. Scale bar, 50 μm. (C and D) Percentage of Ki-67^+^ cells (C) and INS^+^ cells (D) in the mCherry^+^ cell population. Data are shown as mean ± S.E.M. (n = 3 biological replicates, each dot represents cell counts in a single aggregate from one of three independent experiments). (E and F) Cells were sorted based on mCherry at the EN stage, RNA-seq analysis performed (n = 4 biological replicates), and differentially expressed genes in control and poly-miR transduced cells identified. Enriched GO categories (top) and log2-fold change (FC) of exemplary genes (bottom) among genes down- (E, p < 0.05, permutation test) and up-regulated (F, p < 0.05, permutation test) by the poly-miR. *p < 0.05, Student's t test. See also Figure S5 and Table S7.

We next determined whether forced expression of Let-7g, Let-7a, miR-200a, and miR-375 represses cell cycle progression in hESC-derived pancreatic progenitor cells, as predicted by our computational analysis. We transduced PE cells with the poly-miR lentiviral construct and differentiated these cells for another 6 days as 3D aggregates to the early pancreatic endocrine (EN) stage, when insulin^+^ cells are first present (Figure 5A). Sectioned aggregates were then stained for the proliferation marker Ki-67. Consistent with our computational prediction, forced expression of the miRNAs reduced the percentage of Ki-67^+^ cells (Figures 5B and 5C). The miRNAs likely exhibit their anti-proliferative effect in progenitors and not beta cells, as insulin^+^ cells in EN stage cultures were mostly Ki67^−^ in both control vector- and poly-miR-transduced aggregates (Figure S5A).

Since cell cycle exit and endocrine cell differentiation are tightly coupled (Kim et al., 2015, Miyatsuka et al., 2011, Piccand et al., 2014), we tested whether the reduction in Ki-67^+^ cells after miRNA over-expression was associated with an increase in the number of insulin^+^ cells. Indeed, we observed a higher percentage of insulin^+^ cells in aggregates expressing the poly-miR construct compared with vector-transduced aggregates (Figures 5B and 5D). The bias of our culture conditions for the differentiation of insulin^+^ cells (Figure S5B) precluded quantification of other endocrine cell types. To further determine how Let-7g, Let-7a, miR-200a, and miR-375 over-expression affects gene expression at the EN stage, we conducted RNA-seq analysis of sorted mCherry^+^ cells. Consistent with the reduction in Ki67^+^ cells in poly-miR-transduced cultures (Figure 5C), genes associated with cell cycle regulation, such as *CCND1*, *CDK4*, and *PCNA*, were enriched among genes down-regulated (p < 0.05, permutation test) by forced miRNA expression (Figure 5E and Tables S7A and S7B). Up-regulated genes (p < 0.05, permutation test) comprised endocrine cell-characteristic genes involved in the regulation of insulin secretion and ion transport (e.g., *GLPR1*, *SYT1*, *CACNA1A*, *SLC2A1*) (Figure 5F and Tables S7A and S7C), further supporting the conclusion that Let-7g, Let-7a, miR-200a, and miR-375 promote endocrine cell differentiation. We note that insulin mRNA levels were slightly decreased rather than increased in poly-miR transduced cells (Table S7A), suggesting that miRNAs or their target genes could affect insulin protein levels at the posttranscriptional level. Taken together, our data support a model whereby endocrine-enriched Let-7g, Let-7a, miR-200a, and miR-375 are part of a gene regulatory network that triggers cell cycle exit to promote endocrine cell differentiation.

## Discussion

Here, we identified 14 miRNAs (Let-7g, Let-7a, Let-7f, Let-7b, miR-200a, miR-200c, miR-204, miR-99b, miR-141, miR-127, miR-7, miR-27b, miR-98, and miR-375), that are induced during human beta cell differentiation. We further studied five miRNAs (Let-7g, Let-7a, miR-200a, miR-375, and miR-127) with a high fold-change during endocrine cell differentiation and experimentally show that these miRNAs induce cell cycle exit in pancreatic progenitor cells. By constructing an integrated miRNA-gene regulatory network of endocrine cell differentiation, we show that these miRNAs likely contribute to endocrine cell differentiation by directly regulating different sets of cell cycle-associated TFs.

To analyze how islet cell-enriched miRNAs cooperate to drive endocrine cell differentiation, we developed a computational method to model the relationship of miRNAs, TFs, and miRNA-regulated genes. Our computational model builds on a previously published approach for constructing miRNA regulatory networks (Gosline et al., 2016) and integrates chromatin state and expression data to build a multi-layer network. Our approach differs in a few key aspects from published methodologies. First, it incorporates predictions from both CLIP-seq data and TargetScan into a combined score that is assigned to network edges. In addition, our scoring system focuses on a set of miRNAs identified experimentally and weighs the number of miRNAs contributing to each path, accounting for potential synergistic effects of miRNAs on downstream gene expression changes. As such, the algorithm presented here can be applied to other cellular contexts with matching miRNA/mRNA/chromatin data and provides a useful framework for the prediction of miRNA effects.

We found that islet cell-enriched miRNAs Let-7g, Let-7a, miR-200a, miR-375, and miR-127 repress different transcripts involved in cell cycle regulation and therefore might synergize in driving cell cycle exit and endocrine cell differentiation. All four miRNAs have been implicated in the regulation of cell proliferation in other contexts. Like the Let-7 family miRNAs studied here, Let-7b inhibits proliferation and induces neural differentiation when over-expressed in neural progenitors (Zhao et al., 2010). Furthermore, Let-7b, miR-200a, and miR-375 have been shown to induce cell cycle arrest in tumor cells (Liu et al., 2012, Uhlmann et al., 2010, Wang et al., 2011). Likewise, acute over-expression of miR-375 in dedifferentiated beta cells reduces their proliferation and promotes their redifferentiation (Nathan et al., 2015). This anti-proliferative effect of miR-375 is opposite to observations in *miR-375*-deficient mice, which exhibit decreased beta cell proliferation (Poy et al., 2009). Since these mice carry a germline mutation of *miR-375,* it is possible that the observed decrease in beta cell proliferation is the consequence of a developmental defect rather than a reflection of miR-375 directly regulating inhibitors of cell cycle progression.

Pancreatic endocrine cell differentiation is tightly linked to cell cycle exit. In both mice and humans, endocrine cell differentiation depends on the TF NGN3 (encoded by *NEUROG3*) (Gradwohl et al., 2000, McGrath et al., 2015), which commits pancreatic progenitors to the endocrine lineage and promotes cell cycle exit by inducing the cell cycle inhibitors Cdkn1a (p21/CIP1) and Pak3 (Miyatsuka et al., 2011, Piccand et al., 2014). We observed no effect of either combined or individual Let-7g, Let-7a, miR-200a, and miR-375 over-expression on *NEUROG3* mRNA levels (Tables S2A–S2D, S2F), suggesting that these miRNAs exert their effect on proliferation independent of NGN3. However, Let-7g, Let-7a, miR-200a, and miR-375 expression with the poly-miR construct significantly induced the NGN3 target gene and endocrine differentiation factor *NEUROD1* (Ahnfelt-Ronne et al., 2007). Based on our computational model, these miRNAs are predicted to modulate *NEUROD1* expression indirectly through down-regulation of *NEUROD1* upstream TFs. Given that NEUROD1 can promote cell cycle exit through direct activation of *Cdkn1a* (Mutoh et al., 1998), miRNA-mediated modulation of *NEUROD1* levels likely contributes to the observed effect of islet-enriched miRNAs on cell proliferation and differentiation.

Gain- and loss-of-function studies in model organisms have shown that the repressive effects of miRNAs on their targets is mostly modest, which has led to the view that miRNAs act to fine-tune gene expression. Consistent with this view, we observed relatively small effects of miRNA over-expression on gene expression, cell proliferation, and endocrine cell differentiation. However, these results do not mean that the miRNAs are not important for endocrine cell differentiation. We over-expressed islet cell-enriched miRNAs in an *in vitro* system where growth factor conditions have been optimized for efficient beta cell differentiation. Therefore, the miRNAs might not be limiting in the context of these optimized conditions. Studies in model organisms underscore the idea that miRNAs confer robustness to developmental processes and become limiting only under conditions of stress. For example, loss of miR-7 has little effect on *Drosophila* sensory organ development under normal conditions, but when environmental stresses are added to the developing organism, miR-7 becomes necessary (Li et al., 2009). Similar examples exist in worms and mice, where miRNA deletions lead to significant developmental perturbations only on sensitized backgrounds or under stress (Brenner et al., 2010, Chivukula et al., 2014). Further illustrating that miRNAs can have significant biological effects in specific contexts, miRNAs have been shown to drastically augment reprogramming efficiencies (Anokye-Danso et al., 2011, Yoo et al., 2011). Therefore, the here-identified islet cell-enriched miRNAs could help develop still missing protocols for robust direct reprogramming of human endocrine cells.

### Limitations of the Study

One limitation of our approach for identifying endocrine cell differentiation-relevant miRNAs is the focus on miRNAs that repress mRNAs. There is evidence that miRNAs can activate gene expression or directly reduce protein levels (El Ouaamari et al., 2008, Jopling et al., 2008, Vasudevan et al., 2007). It is possible that some of the identified miRNAs regulate endocrine cell differentiation through these mechanisms. Another limitation is that we compared miRNA profiles in pancreatic progenitors and mature human endocrine cells. Therefore, we do not know how these miRNAs are regulated during postnatal endocrine cell maturation. Finally, our network modeling approach predicts synergist effects of Let-7g, Let-7a, miR-200a, and miR-375 on cell cycle regulation. This predicted synergy will have to be experimentally validated.

## Methods

All methods can be found in the accompanying Transparent Methods supplemental file.

## Data and Code Availability

All RNA-seq and ATAC-seq data generated in this study can be found at GEO with accession number GSE115327.

Accession numbers for additional data used in this study are as follows: GSE52314 (small RNA-seq, sorted alpha and beta cells); GSE51924 (CLIP-seq, human islets); E-MTAB-1086 (RNA-seq, PE cells); GSE54471 (H3K27ac, H3K4me3 ChIP-seq, PE cells); GSE51311, E-MTAB-1919, E-MTAB-189, and E-MTAB-191 (H3K27ac, H3K4me3 ChIP-seq, human islets).

## Supplemental Information

Document S1. Transparent Methods and Figures S1–S5

Table S1. miRNA Expression in PE, Pancreatic Alpha and Beta Cells, Table S1 Is Associated with Figure 1 (A) miRNA expression values (RPM) in PE, pancreatic alpha and beta cells. (B) miRNA expression fold-change in beta cells versus PE. (C) miRNA expression fold-change in alpha cells versus PE.

Table S2. Genes Regulated by Individual miRNAs and poly-miR in PE Cells, Table S2 Is Associated with Figures 2 and 3 (A) Let-7a, (B) Let-7g, (C) miR-200a, (D) miR-375, (E) miR-127, and (F) poly-miR regulated genes. (G) poly-miR repressed genes also decreased in islet versus PE.

Table S3. GO Analysis of Individual miRNA- and poly-miR-repressed Genes, Table S3 Is Associated with Figures 2 and 3 GO analysis of genes that decrease in expression in islet compared with PE and are repressed by (A) Let-7a, (B) Let-7g, (C) miR-200a, (D) miR-375, (E) miR-127, and (F) poly-miR.

Table S4. Analysis of Predicted Direct Targets for poly-miR, Table S4 Is Associated with Figure 3 (A) List of poly-miR predicted direct targets. (B) GO analysis of poly-miR predicted direct targets. (C) Poly-miR predicted direct targets with lower expression in islet than PE.

Table S5. GO Analysis of All Genes in the miRNA-Regulated Network, Table S5 Is Associated with Figure 4

Table S6. Network-Based Ranking of Cell Cycle Genes, Table S6 Is Associated with Figure 4

Table S7. Genes Regulated by poly-miR at EN Stage, Table S7 Is Associated with Figure 5 (A) poly-miR regulated genes. GO analysis of genes that are repressed (B) and induced (C) by poly-miR.
