# Supplementary material for: A Network of microRNAs Acts to Promote Cell Cycle Exit and Differentiation of Human Pancreatic Endocrine Cells
Source: iScience. 2019 Nov 1;21:681–94. doi: 10.1016/j.isci.2019.10.063 (PMC6889369; doi:10.1016/j.isci.2019.10.063)
Supplement: Document S1. Transparent Methods and Figures S1–S5 [file mmc1.pdf]

ISCI, Volume *21*

Supplemental Information

A Network of microRNAs Acts to Promote Cell Cycle Exit and Differentiation of Human Pancreatic Endocrine Cells

Wen Jin, Francesca Mulas, Bjoern Gaertner, Yinghui Sui, Jinzhao Wang, Ileana Matta, Chun Zeng, Nicholas Vinckier, Allen Wang, Kim-Vy Nguyen-Ngoc, Joshua Chiou, Klaus H. Kaestner, Kelly A. Frazer, Andrea C. Carrano, Hung-Ping Shih, and Maike Sander

Supplemental figures and legends

Figure S1

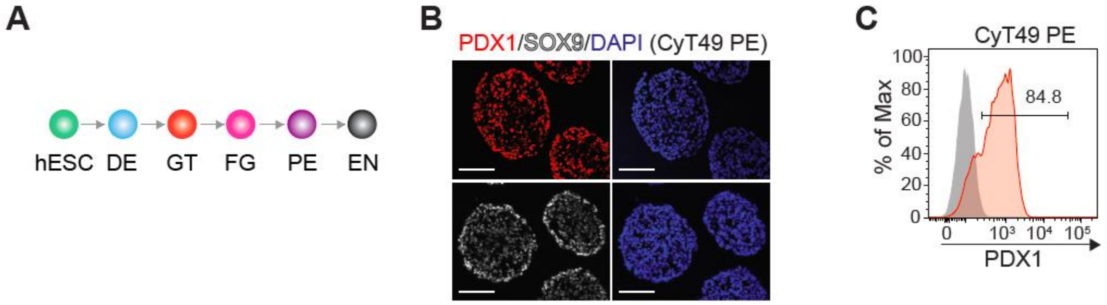

Figure S1: Pancreatic endoderm differentiated from CyT49 hESCs. Related to Figure 1.
(A) Schematic of the hESC-based differentiation strategy. **(B)** Immunofluorescence staining of PE cell aggregate sections for PE-specific markers PDX1 and SOX9. Scale bar, 50 µm. **(C)** Representative flow cytometry analysis at PE stage for PDX1. hESC, human embryonic stem cells; DE, definitive endoderm; GT, gut tube; FG posterior foregut; PE, pancreatic endoderm; EN, endocrine cell stage.

Figure S2

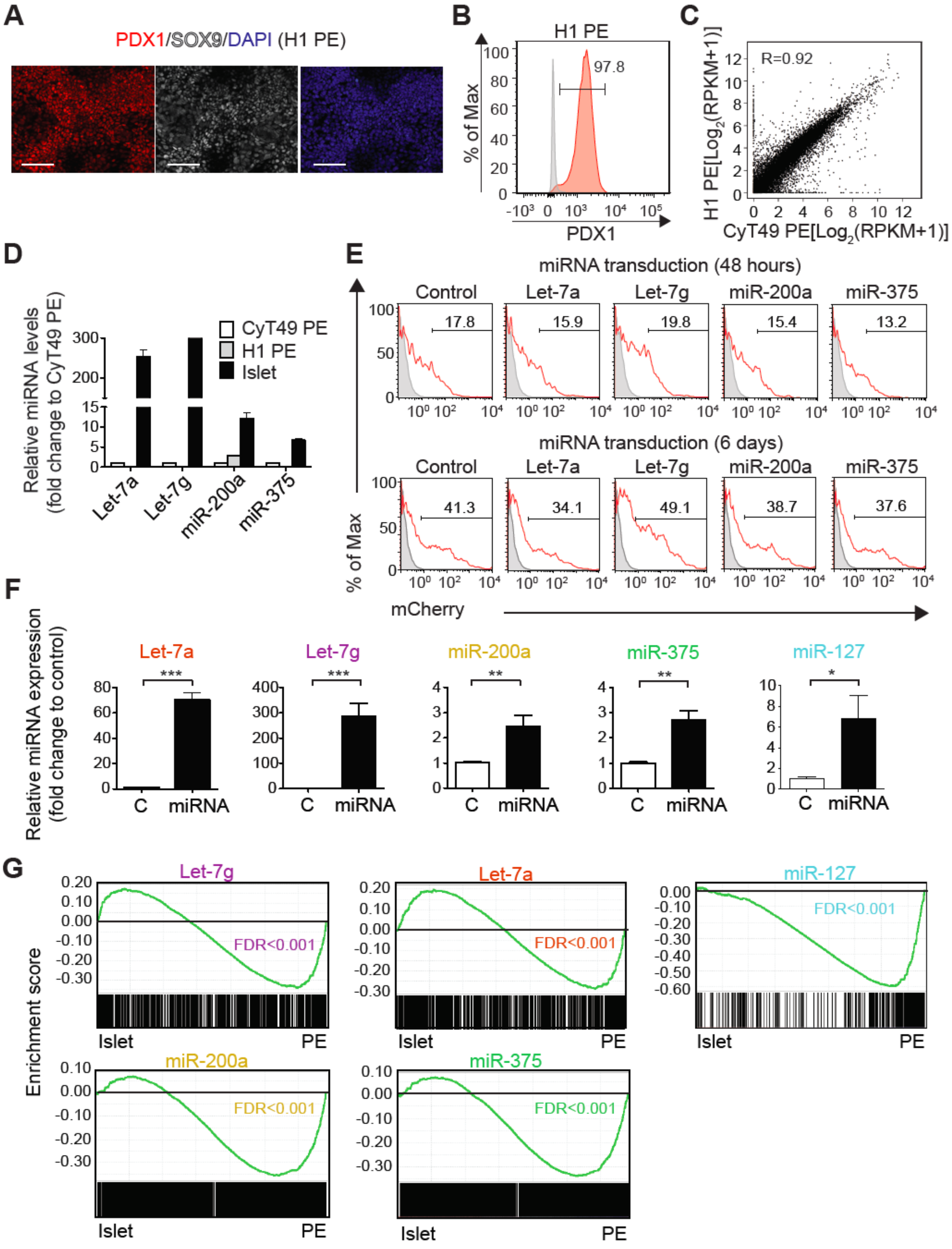
A
PDX1/SOX9/DAPI (H1 PE)
B
H1 PE
97.8
% of Max
PDX1
C
R=0.92
H1 PE[Log2(RPKM+1)]
CyT49 PE[Log2(RPKM+1)]
D
Relative miRNA levels (fold change to CyT49 PE)
CyT49 PE
H1 PE
Islet
Let-7a
Let-7g
miR-200a
miR-375
E
miRNA transduction (48 hours)
Control
Let-7a
Let-7g
miR-200a
miR-375
17.8
15.9
19.8
15.4
13.2
miRNA transduction (6 days)
41.3
34.1
49.1
38.7
37.6
% of Max
mCherry
F
Relative miRNA expression (fold change to control)
Let-7a
Let-7g
miR-200a
miR-375
miR-127
C
miRNA
G
Enrichment score
Let-7g
Let-7a
miR-127
miR-200a
miR-375
FDR<0.001
Islet
PE

Figure S2. Forced expression of individual miRNAs in hESC-derived pancreatic progenitor cells. Related to Figure 2.

(A) Immunofluorescence staining of pancreatic endoderm (PE) differentiated from H1 hESCs for PDX1 and SOX9. Scale bar, 50 µm. **(B)** Representative flow cytometry analysis at PE stage for PDX1. **(C)** Scatter plot showing correlation in mRNA expression between PE cells derived from H1 hESCs and CyT49 hESCs. **(D)** Expression of indicated miRNAs in H1-derived PE cells and islets relative to CyT49-derived PE cells determined by Taqman qPCR. Data are shown as mean ± S.E.M. (n = 3 technical replicates). **(E)** Representative flow cytometry analysis for mCherry 48 h (top row) and 6 days (bottom row) after lentiviral transduction with miRNA-mCherry constructs. Gating for cell sorting is shown. **(F)** Relative expression of indicated miRNAs determined by Taqman qPCR in H1 PE cells 48 h after lentiviral transduction with miRNAs or vector control (C). Data are shown as mean ± S.E.M. (n = 3 biological replicates). ***P* < 0.01, ****P* < 0.001; Student's t-test. **(G)** GSEA plots showing enrichment of genes repressed by Let-7g, Let-7a, miR-200a, miR-375, and miR-127 in islets (n = 3) compared to PE (n = 2). False Discovery Rate (FDR) is shown.

Figure S3

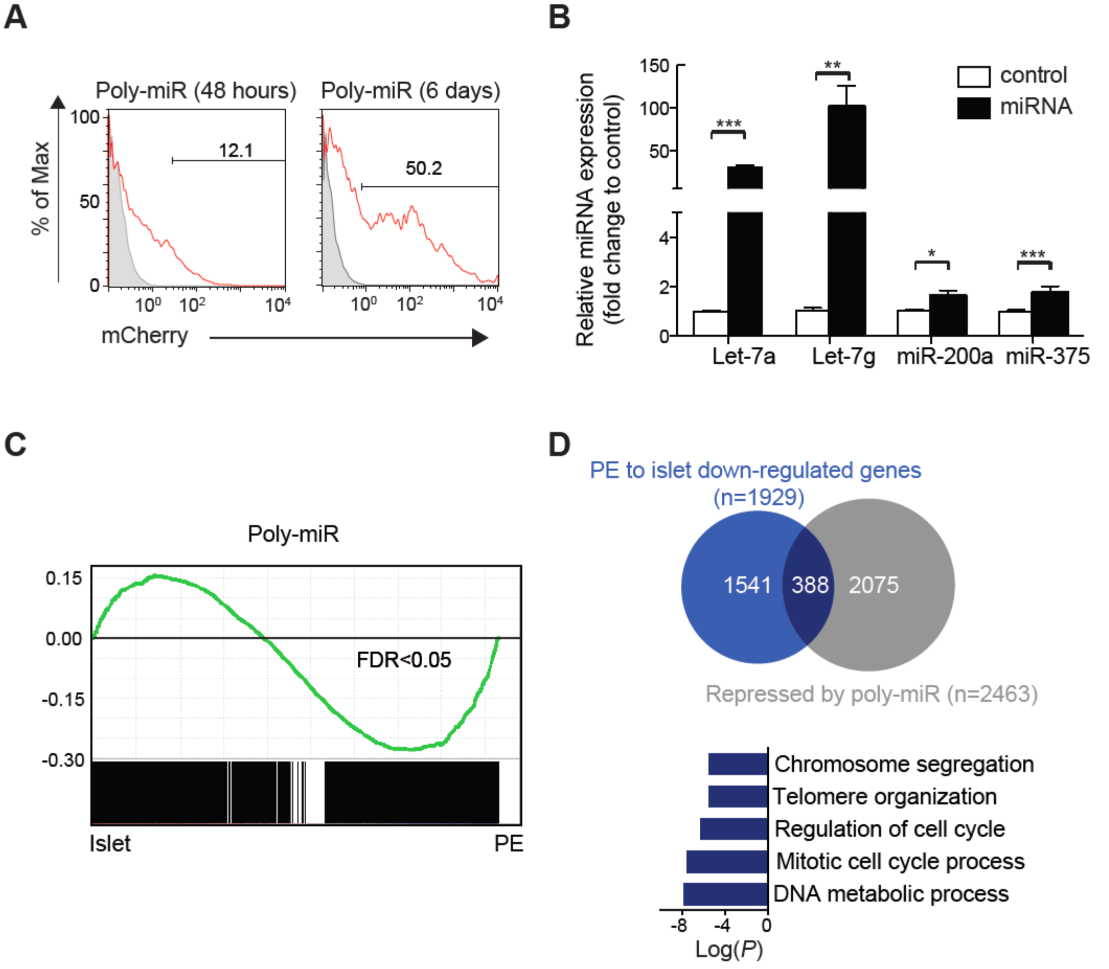

Figure S3. Forced expression of a polycistronic construct for four miRNAs in hESC-derived pancreatic progenitor cells. Related to Figure 3.
(A) Representative flow cytometry analysis for mCherry 48 h (left) and 6 days (right) after lentiviral transduction with poly-miR-mCherry construct. Gating for cell sorting is shown. (**B)** Relative expression of indicated miRNAs determined by Taqman qPCR in H1 PE cells 48 h after lentiviral transduction with a vector control or a polycistronic construct containing Let-7g, Let-7a, miR-200a, and miR-375 (poly-miR). Data are shown as mean ± S.E.M. (n = 3 biological replicates). ***P* < 0.01, ****P* < 0.001; Student's t-test. (**C)** GSEA plot showing enrichment of genes repressed by the poly-miR construct in islets compared to PE. False Discovery Rate (FDR) is shown. **(D)** Venn diagram showing the overlap between genes down-regulated in islets compared to PE (blue) and genes repressed by the poly-miR construct (grey). Top five GO categories enriched among genes repressed by the poly-miR construct and down-regulated in islets compared to PE are shown on the bottom.

Figure S4

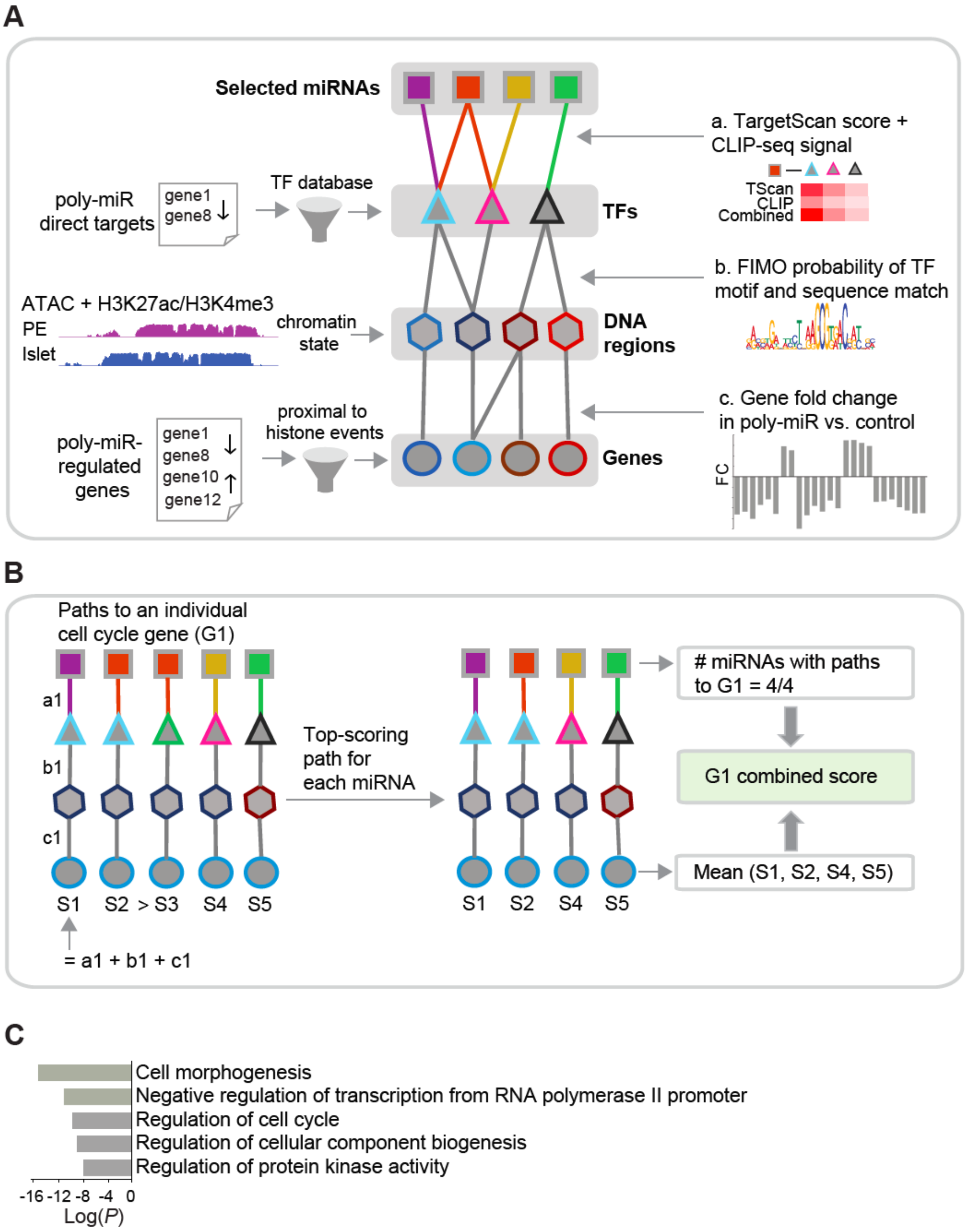
A
Selected miRNAs
a. TargetScan score +
CLIP-seq signal
TScan
CLIP
Combined
poly-miR
direct targets
gene1
gene8
TF database
TFs
b. FIMO probability of TF
motif and sequence match
ATAC + H3K27ac/H3K4me3
PE
Islet
chromatin
state
DNA
regions
c. Gene fold change
in poly-miR vs. control
FC
poly-miR-
regulated
genes
gene1
gene8
gene10
gene12
proximal to
histone events
Genes
B
Paths to an individual
cell cycle gene (G1)
a1
b1
c1
S1
S2 > S3
S4
S5
= a1 + b1 + c1
Top-scoring
path for
each miRNA
S1
S2
S4
S5
miRNAs with paths
to G1 = 4/4
G1 combined score
Mean (S1, S2, S4, S5)
C
Cell morphogenesis
Negative regulation of transcription from RNA polymerase II promoter
Regulation of cell cycle
Regulation of cellular component biogenesis
Regulation of protein kinase activity
-16 -12 -8 -4 0
Log(P)

Figure S4. Approach to identify core network of miRNA-regulated genes in pancreatic progenitor cells. Related to Figure 4.

(A,B) Schematic of approach to identify core network of miRNA-regulated transcription factors and cell cycle genes. Building of network **(A)** and probing of network **(B)** is shown. The nodes of the graph represent miRNAs [squares; Let-7g (purple), Let-7a (red), miR-200a (yellow), and miR-375 (green)], transcription factors (triangles), transcription factor binding regions (hexagons) and genes (circles). In **(A)**, the source for each node is indicated on the left, while evidence (indicated by *a, b,* and *c*) used to calculate scores is indicated on the right. In **(B)**, path-based scoring of an individual gene [e.g. gene 1 (G1)] is shown. See Materials and Methods for details. **(C)** Top five GO categories enriched in genes comprising the miRNA-regulated core network in **(A)**. TF, transcription factor; G, gene; S, score.

Figure S5

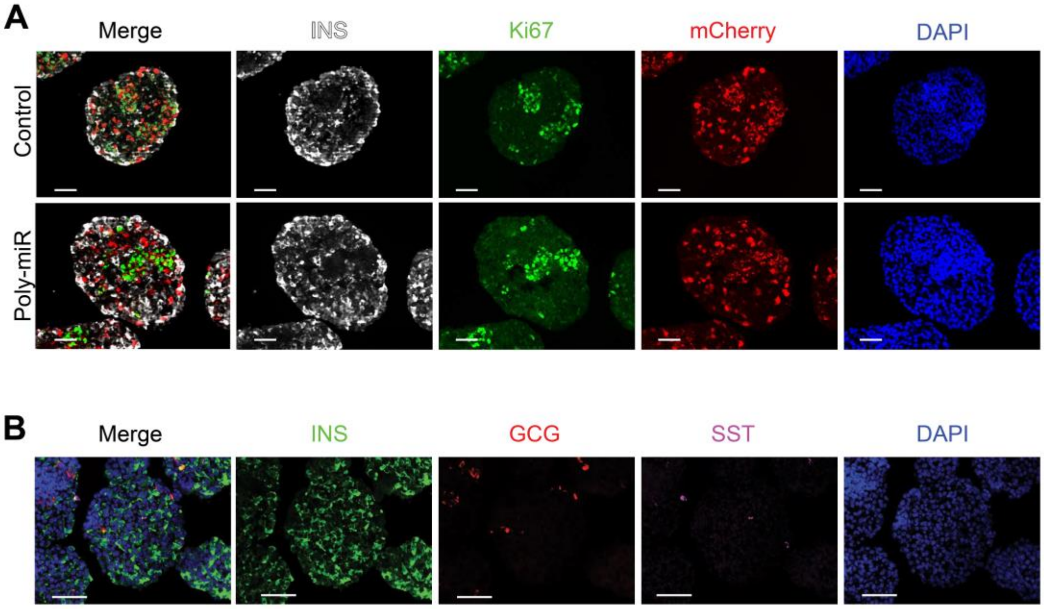

Figure S5. Endocrine cell differentiation from H1 hESCs. Related to Figure 5. **(A)** Representative images showing immunofluorescence staining for insulin (INS), Ki-67, mCherry, and DAPI at the endocrine cell (EN) stage for control vector (top) or poly-miR (bottom) transduced aggregates. **(B)** Immunofluorescence staining of sections from EN stage aggregates for insulin (INS), glucagon (GCG), and somatostatin (SST). Scale bar, 50 µm.

Transparent Methods

Contact for reagent and resource sharing

Further information and requests for reagents may be directed and will be fulfilled by the corresponding author Maike Sander (masander@ucsd.edu).

Experimental model and subject details

Human islets

Human cadaveric pancreatic islets for the Taqman miRNA analysis (Donor ID:1-3) and for the RNA-seq and ATAC-seq analysis (Donor ID: 4-7) were obtained through the Integrated Islet Distribution Program (IIDP). The islets had ≥ 90% purity and ≥ 90% viability. Upon receipt, islets were handpicked and immediately processed for RNA extraction or isolation of nuclei.

Donor ID	Donor Age	Donor Sex	Dia-betes*	BMI	Race	Cause of Death
1	53	Male	No	27.2	Caucasian	CVA/STRO-KE
2	48	Female	No	31.0	American Indian or Alaska Native	CVA/STRO-KE
3	48	Male	No	27.7	Caucasian	CVA/STROKE
4	55	Male	No	29.8	Black or African American	CVA/STRO-KE
5	59	Female	No	24.7	Caucasian	CNS tumor
6	55	Male	No	23.2	Caucasian	Head trauma
7	56	Female	No	33.4	Black or African American	CVA/STRO-KE

*Diabetes status was defined by the patient’s medical record and, when available, hemoglobin A1c levels.

Maintenance and differentiation of H1 hESCs

hESC research was approved by the University of California, San Diego, Institutional Review Board and Embryonic Stem Cell Research Oversight Committee. All hESC experiments were performed in H1 hESCs with the exception of miRNA expression profiling, for which CyT49 hESCs were used.

H1 hESCs were maintained and differentiated as described with some modifications (Rezania et al., 2014). In brief, hESCs were cultured in mTeSR1 media (Stem Cell Technologies) and propagated by passaging cells every 3 to 4 days using Accutase (eBioscience) for enzymatic cell dissociation. For differentiation of H1 cells, we employed a 2D monolayer culture format up to day 11 of differentiation. Cells were then dissociated using accutase for 10 min, reaggregated by plating the cells in a low attachment 6-well plate on an orbital shaker (100 rpm) in a 37 °C incubator. Cells were subsequently cultured in suspension from Days 11-14.

On Day 0, dissociated hESCs were resuspended in mTeSR1 media (see media compositions below) and seeded onto Matrigel-coated 12-well plates by adding 1 ml of cell suspension (~8 x 10^5 cells/well) to each well. The following day, undifferentiated cells were washed in stage 1 medium and then differentiated using a multi-step protocol with stage-specific media (see below) and daily media changes.

All stage-specific base media were comprised of MCDB 131 medium (Thermo Fisher Scientific) supplemented with $NaHCO_3$, GlutaMAX, D-Glucose, and BSA using the following concentrations:

Stage 1/2 medium: MCDB 131 medium, 1.5 g/L NaHCO3, 1X GlutaMAX, 10 mM D-Glucose, 0.5% BSA

Stage 3/4 medium: MCDB 131 medium, 2.5 g/L NaHCO3, 1X GlutaMAX, 10 mM D-glucose, 2% BSA

Stage 5 medium: MCDB 131 medium, 1.5 g/L NaHCO3, 1X GlutaMAX, 20 mM D-glucose, 2% BSA

Media compositions for each stage were as follows:

Stage 1 (Days 0-2): base medium, 100 ng/ml Activin A, 25 ng/ml Wnt3a (Day 0). Day 1-2: base medium, 100 ng/ml Activin A

Stage 2 (Days 3-5): base medium, 0.25 mM L-Ascorbic Acid (Vitamin C), 50 ng/mL FGF7

Stage 3 (Days 6-7): base medium, 0.25 mM L-Ascorbic Acid, 50 ng/mL FGF7, 0.25 µM SANT-1, 1 µM Retinoic Acid, 100 nM LDN193189, 1:200 ITS-X, 200 nM TPB

Stage 4 (Days 8-10): base medium, 0.25 mM L-Ascorbic Acid, 2 ng/mL FGF7, 0.25 µM SANT-1, 0.1 µM Retinoic Acid, 200 nM LDN193189, 1:200 ITS-X, 100nM TPB

Stage 5 (Days 11-14): base medium, 0.25 µM SANT-1, 0.05 µM Retinoic Acid, 100 nM LDN193189, 1:200 ITS-X, 1 µM T3, 10 µM ALK5 inhibitor II, 10 µM ZnSO4, and 10 µg/mL Heparin, 10 µM ROCK inhibitor

End of stage 1 = definitive endoderm

End of stage 2 = gut tube

End of stage 3 = posterior foregut

End of stage 4 = pancreatic endoderm

End of stage 5 = endocrine cells

Maintenance and differentiation of CyT49 hESCs

CyT49 hESCs were maintained and differentiated as described (Xie et al., 2013). Propagation of CyT49 hESCs was carried out by passing cells every 3 to 4 days using Accutase™ (eBioscience) for enzymatic cell dissociation, and with 10% (v/v) human AB serum (Valley Biomedical) included in the hESC medium the day of passage. hESCs were seeded into tissue culture flasks at a density of 50,000 cells/cm2.

CyT49 hESC medium was comprised of DMEM/F12 (Corning; 45000-346) supplemented with 10% (v/v) KnockOut™ Serum Replacement (Thermo Fisher Scientific), 1X MEM non-essential amino acids (Thermo Fisher Scientific), 1X GlutaMAX™ (Thermo Fisher Scientific), 1% (v/v) penicillin-streptomycin (Thermo Fisher Scientific), 0.1mM 2-mercaptoethanol (Thermo Fisher Scientific), 10ng/mL Activin A (R&D Systems), and 10ng/mL Heregulin-β1 (PeproTech).

Pancreatic differentiation of CyT49 hESCs was performed as previously described (Schulz et al., 2012). Briefly, we employed a suspension-based format using rotational culture. Undifferentiated hESCs were aggregated by preparing a single-cell suspension in hESC media at 1×10^6 cells/mL and overnight culture in six-well ultra-low attachment plates (Costar) with 5.5ml per well on an orbital rotator (Innova2000, New Brunswick Scientific) at 95 rpm. The following day, undifferentiated aggregates were washed in RPMI media (Corning) and then differentiated using a multistep protocol with daily media changes and continued orbital rotation at either 95 rpm or at 105 rpm on Days 4 to 8.

Stage 1/2 medium: RPMI medium (Corning), 0.2 % (vol/vol) FBS, 1X GlutaMAX

Stage 3/4 medium: DMEM High Glucose medium (HyClone), 0.5X B-27 Supplement, 1X GlutaMAX

Media compositions for each stage were as follows:

Stage 1 (Days 0-1): Day 0: RPMI/FBS, 100ng/mL Activin A, 50ng/mL mouse Wnt3a, 1:5000 ITS. Day 1: RPMI/FBS, 100 ng/mL Activin A, 1:5000 ITS

Stage 2 (Days 2-4): Day 2: RPMI/FBS, 2.5μM TGFβ R1 kinase inhibitor IV, 25ng/mL KGF, 1:1000 ITS. Days 3-4: RPMI/FBS, 25ng/mL KGF, 1:1000 ITS

Stage 3 (Days 5 -7): DMEM/B27, 3nM TTNPB, 0.25mM KAAD-Cyclopamine, 50ng/mL Noggin

Stage 4 (Days 7-10): DMEM/B27, 50ng/mL KGF, 50ng/mL EGF

End of stage 1 = definitive endoderm

End of stage 2 = gut tube

End of stage 3 = posterior foregut

End of stage 4 = pancreatic endoderm

Cell line

HEK293T cells were maintained in DMEM containing 100 units/mL penicillin and 100 mg/mL streptomycin sulfate supplemented with 10% fetal bovine serum (FBS).

Method details

Immunocytochemistry

Cells were washed twice before fixation with 4% paraformaldehyde in PBS for either 30 min at room temperature, or overnight at 4°C. Cells were then washed three times with PBS and incubated in 30% sucrose at 4°C overnight before mounting in Optimal Cutting Temperature Compound (Tissue-Tek) and sectioning at 10 µm. Immunocytochemistry was performed as described (Xie et al., 2013). The following primary antibodies and dilutions were used: guinea pig anti-PDX1 (gift from Dr. Christopher Wright, Vanderbilt University) 1:1000; rabbit anti-SOX9 (Millipore, AB5535) 1:1000; rabbit anti-Ki-67 (ThermoFisher, RM-9106-S1) 1:200; guinea pig anti-insulin (LINCO, 4011-01) 1:1000. Secondary antibodies were Cy5-, Cy3-, or Alex488-conjugated donkey antibodies against guinea pig or rabbit (Jackson Immuno Research Laboratories). Images were

acquired on a Zeiss Axio-Observer-Z1 microscope with a Zeiss AxioCam digital camera and figures prepared with Adobe Photoshop CS6/Illustrator CS5.

To determine the percentage of Ki-67^{+} cells in the mCherry^{+} cell population, at least ten sections from different aggregates were analyzed per hPSC differentiation. For each condition, three independent hPSC differentiations were performed. Ki-67^{+} and mCherry^{+} cells were quantified using HALO software (PerkinElmer Inc).

Fluorescence-activated cell sorting and intracellular flow cytometry

hESC-derived PE cells were dissociated to a single-cell suspension with Accutase (Stemcell Technologies) at 37°C for 10 min. Accutase was neutralized with FACS sorting buffer [1% (wt/vol) FBS, 1 mM EDTA, 25mM Hepes, PBS]. FACS was performed on a FACS Fortessa equipped with FACS DiVa software (BD Biosciences). Cells were sorted into Trizol for RNA analysis. For intracellular flow cytometry, dissociated cells were fixed, permeabilized with BD Cytoperm/Cytofix (BD Bioscience), and stained with anti-PDX1-PE conjugated antibody (BD Biosciences, 562161; 1:20) at room temperature for 30 min, washed, and resuspended in FACS buffer. Flow cytometry analysis was performed on FACSCanto II (BD Biosciences) and analyzed with FlowJo software (FlowJo LLC).

TaqMan microRNA assay

qRT-PCR for miRNAs was performed using the TaqMan MicroRNA Reverse Transcription Kit (Applied Biosystems, Cat. No. 4366596). Briefly, 10 ng of total RNA was reverse transcribed using RT primers from the TaqMan MicroRNA Assay kit [Applied Biosystems; probe catalogue numbers: Let-7a (000377), Let-7g (002282), miR-127 (000452), miR-200a (000502), miR-375 (000564), miR-7 (000268), miR-99b (000436), and RUN44 (001094). qRT-PCR was performed on a Bio-rad CFX96 real-time system using the TaqMan Universal PCR Master Mix (Applied Biosystems, Cat. No. 4324018) and TaqMan probes from the TaqMan MicroRNA Assay kit. miRNA levels were determined on three independent samples and values were normalized to endogenous snoRNA RNU44.

miRNA expression vector construction

To generate miRNA expression vectors, 270 nt of the miRNA gene primary transcript, including the 22 nt mature miRNA and 125 nt of genomic sequence flanking each side of the miRNA (Chen et al., 2004), were amplified. Let-7a and Let-7g were expressed with mutations in their loop sequence to block LIN28 binding and ensure proper miRNA processing (Piskounova et al., 2008). For transduction of PE cells, a modified version of pLKO.3G was used, in which GFP was exchanged for mCherry (pLKO.mcherry). For the polycistronic miRNA expression vector, a gBlock gene fragment encompassing miR-375, Let-7a, Let-7g, and miR-200a was cloned into pLKO.mcherry.

Lentivirus production and transduction of PE cells

High-titer lentiviral supernatants were generated by co-transfection of the miRNA expression vector and the lentiviral packaging construct into HEK293T cells as described (Xie et al., 2013). Briefly, miRNA expression vectors were cotransfected with the pCMV-R8.74 (Addgene, #22036) and pMD2.G (Addgene, #12259) expression plasmids into HEK293T cells using a 1mg/ml PEI solution (Polysciences). Lentiviral supernatants were collected at 48 hr and 72 hr after transfection. Lentiviruses were concentrated by ultracentrifugation for 120 min at 19,500 rpm using a Beckman SW28 ultracentrifuge rotor at 4°C. The titer routinely achieved was $5*10^8$~10^9 TU/ml. For PE cell transductions, H1 hESCs were differentiated to the PE stage (Day 8 of differentiation) in monolayer cultures and transduced with lentivirus at a MOI of 2. For RNA analysis, cells were collected 48 hr after transduction.

Small RNA sequencing and data analysis

Small RNA-seq data from sorted human alpha and beta cells have been described (Kameswaran et al., 2014). RNA from PE stage CyT49 hESC cultures was isolated using the miRVana miRNA Isolation kit (Thermo Fisher Scientific). 3 µg of RNA was used for library preparation using the TruSeq Small RNA sample preparation kit (Illumina) and a Pippin Prep (Sage Science) for size selection with a 3% cassette (CSD3010). RNA was prepared for sequencing using the Illumina protocol (Illumina FC-102-1009) and amplified libraries were sequenced on an Illumina Genome Analyzer II (Illumina FC-104-1003). Sequenced libraries were

processed as described (Kameswaran et al., 2014). miRNAs with sample values below 1 RPM were excluded from the analysis. There was one replicate each for hESC-derived PE, alpha, and beta cells. Each miRNA expression value was log_2-transformed and displayed in a heatmap.

RNA sequencing, mapping and data analysis

RNA quality was assessed using TapeStation (Agilent Technologies). Libraries were prepared according to the instructions of Illumina's TruSeq RNA library prep kit. Libraries were quantified using High Sensitivity DNA screen tape (Agilent Technologies) and Qubit dsDNA High Sensitivity (Life Technologies) assays. Finally, libraries were multiplexed and sequenced on a HiSeq 2500 (Illumina) sequencer using single-end sequencing.

RNA-seq samples were mapped to the UCSC human transcriptome (hg19/GRCh37) by the Spliced Transcripts Alignment to a Reference (STAR) aligner (STAR-STAR_2.4.0f1), allowing for up to 10 mismatches (Dobin et al., 2013). Only reads aligned uniquely to one genomic location were retained for subsequent analysis. Expression levels of all genes were quantified by Cufflink (https://github.com/cole-trapnell-lab/cufflinks) using only reads with exact matches. Genes with average RPKM above 1 were retained for further analyses.

Differentially expressed genes were identified using a permutation test, with the number of permutations set to 1000. Briefly, all the samples were shuffled, fold changes were computed to obtain a null distribution, and a *P*-value was estimated for each gene's fold change as a cumulative probability from the

null distribution. For comparison of PE and islet data and poly-miR versus control data at the EN stage, batch effects were removed using ComBat (Johnson et al., 2007).

Gene Set Enrichment Analysis (GSEA) and Gene Ontology (GO) analysis

We applied GSEA (http://www.broad.mit.edu/gsea), which scores a-priori defined gene sets in two different conditions (Subramanian et al., 2005). GSEA (http://www.broad.mit.edu/gsea) was run with the number of permutations for *P*-value computation set to 1000. We used genes significantly repressed by miRNAs ($P < 0.05$, permutation test) as gene sets to determine coordinated regulation in islets compared to PE samples. Gene sets with a false discovery rate of < 0.05 were considered significantly enriched. Enrichment of gene sets for Gene Ontology (GO) terms was tested using Metascape (Tripathi et al., 2015).

ATAC-seq sample preparation

Roughly 50,000 PE or primary human islet cells were used for each ATAC-seq assay as described (Buenrostro et al., 2013). Briefly, cell nuclei were isolated using cold lysis buffer (10 mM Tris-HCl, pH 7.4, 10 mM NaCl, 3 mM MgCl2 and 0.1% IGEPAL CA-630). The nuclei pellet was resuspended in the transposase reaction mix; 25 μL 2x TD buffer, 2.5 μL transposase (Illumina) and 22.5 μL nuclease-free water at 37°C for 30 min. Then transposed DNA fragments were purified using the Qiagen MinElute kit and amplified 10-12 cycles using the

Nextera (Illumina) PCR primers. Libraries were sequenced on HiSeq4000 platform.

ChIP-seq and ATAC-seq data analysis

ChIP-seq and ATAC-seq reads were mapped to the human genome (hg19/GRCh37) using Bowtie (Langmead et al., 2009) and BWA (Li and Durbin, 2009), respectively, and visualized using the UCSC Genome Browser (Kent et al., 2002). Unmapped reads were discarded. After mapping, SAMtools (Li et al., 2009) was used to remove duplicate sequences and merge samples. Here, “SAMtools view -Sbq 30” was used to filter out reads with mapping quality less than 30, "SAMtools rmdup” was used to remove duplicated reads, and "SAMtools merge” was used to merge files of the same histone marker or input condition. ChIP-seq and ATAC-seq analysis was performed in two biological replicates for PE and 4-5 donors for islet. The Pearson correlation among biological replicates ranged from 64% to 96% for human islets and 91% to 96% for PE.

HOMER (Heinz et al., 2010) as used to call ChIP-seq peaks using “findPeaks function” with “–style histone” to call peaks. Stage- and condition-matched input DNA controls were used as background when calling peaks. MACS2 (Zhang et al., 2008) was used to call peaks from ATAC-seq data, with parameters “shift set to 100 bps, smoothing window of 200 bps” and with “nolambda” and “nomodel” flags on.

To link changes in chromatin to gene expression changes, we first defined differential H3K27ac and H3K4me3 peaks in PE and islet (adjusted $P<0.05$,

“getDifferentialPeaksReplicates” function in HOMER) and then used BEDtools (Quinlan and Hall, 2010) to identify overlapping ATAC peaks in PE or islet using a ± 1.5 kb window from the summit of the ATAC peak. Next, we identified the nearest TSS within a 10kb window of the H3K27ac or H3K4me3 peak. We then assessed the concordance of the directionality of changes in gene expression and histone marks by evaluating whether genes near regions showing gain or loss of H3K27ac or H3K4me3 in PE versus islet exhibit significant concordant expression changes (Mann-Whitney test).

Network building

CLIP-seq signal, mRNA expression, ATAC-seq and ChIP-seq binding data were encoded in a graphical model depicted in Figures 4 and Figure S4 by adapting a previously published algorithm (Gosline et al., 2016). Nodes arranged in four different layers, corresponding to miRNAs, transcription factors (TFs), DNA regions, and genes, were identified and connected as follows: For each of the four selected miRNAs, predicted target genes were retrieved through the TargetScan repository (http://www.targetscan.org/vert_71/). For the pairs of miRNA-target gene identified, the corresponding CLIP-seq signal was collected from previously published data (Kameswaran et al., 2014). Among the targets, TFs in the second layer of the network were selected based on down-regulation by poly-miR transfection compared to control with $P < 0.05$ and with an annotation in the TF Animal Database (Zhang et al., 2015). In the third layer of the network, we selected DNA regions showing significant changes in PE versus

islet for H3K27ac or H3K4me3 (see previous paragraph) with the nearest gene showing down-regulation by poly-miR transfection, hereafter referred to as *Rsel*. The *Rsel* DNA regions were filtered for links to the selected TFs by scoring the match of their binding motifs with the DNA regions in the network. Briefly, motifs of selected TFs were extracted from a collection of databases, including JASPAR (http://jaspar.genereg.net/cgi-bin/jaspar_db.pl), Hocomoco (http://hocomoco11.autosome.ru/), and ENCODE-related data sets (Aylward et al., 2018) and scored for matches with narrow regions spanning 300bp around the peak summits of each *Rsel*. Log-odds scores and corresponding *P*-values were obtained using the MEME Suite tool FIMO (http://meme-suite.org/tools/fimo) with default parameters. The last layer was defined by considering genes proximal to DNA regions, as described above, and filtering for those differentially regulated in poly-miR versus control ($P < 0.05$).

A score representing the strength of the association was computed for each pair of connected nodes in the different network layers, as follows: Given the TargetScan context++ score S_i (Agarwal et al., 2015) and the CLIP-seq signal S_j of each miRNA-TF association, their values were normalized in a 0-1 range and combined as $a = (1 - S_i)(1 - S_j)$ (Szklarczyk et al., 2015). Scores of edges connecting TFs to *Rsel* regions were defined as the $b = 1 - q$, q being the q-value returned by FIMO, representing the probability of obtaining the log-odds ratio scores of the matches by chance. Scores from DNA regions to genes were defined as the absolute value of the Log2 Fold Change of each gene x in poly-miR versus control data: $c = |Log2FC(x)|$ A combined score was computed for

each possible path in the network starting from a miRNA to a gene, by adding the contribution of the different layers, as: $S = a + b + c$.

Network-based gene ranking

Given an individual gene, G1, all network paths connecting a miRNA to G1 were considered with their corresponding scores and compared for gene ranking as follows: Among the paths connecting the same miRNA to G1, only the one with the highest score was retained, obtaining a score S_i for each of the *k* miRNAs showing an indirect link to G1, k<=N, with N equal to the number of miRNAs in the network (N=4). A first score summarizing the strength of the association of these retrieved network paths was computed as $S_{net}(G1) = mean(S_i)$, *i* ranging from one to *k*. A second score, accounting for the synergistic effect of several miRNAs on the same gene, was computed as proportion of miRNAs in at least one network path linking to the selected gene G1: $S_{mir}(G1) = k/N$. Finally, a combined score for G1 was obtained as a weighted sum of S_{net} and S_{mir}, i.e. $S_{comb}(G_1) = w * S_{net}(G1) + w * S_{mir}(G1)$, with *w* set to 0.5. This procedure was applied to score individual genes annotated to cell cycle regulation in the Gene Ontology (GO:0051726) and genes were ranked based on S_{comb} values.

Quantification and statistical analysis

Statistical parameters including FDR, R, and *P*-values are reported in the Figures and the Figure Legends.

KEY RESOURCES TABLE

REAGENT or RESOURCE	SOURCE	IDENTIFIER
**Antibodies**		
Rabbit anti-PDX1	Abcam	Cat# ab47267, RRID:AB_777179
Rabbit anti-SOX9	Millipore	Cat# AB5535, RRID:AB_2239761
Guinea pig anti-INS	Dako	Cat# A0564, RRID:AB_10013624
Mouse anti-GCG	Sigma-Aldrich	Cat# G2654, RRID:AB_259852
Rabbit anti-SST	Agilent	Cat# A056601-2
Mouse anti-Ki67	Lab Vision	Cat# RM-9106-S1, RRID:AB_149792
Mouse anti-PDX1, PE conjugate	BD Biosciences	Cat# 562161, RRID:AB_10893589
Mouse IgG1, kappa isotype control (PE conjugate)	BD Biosciences	Cat# 556650, RRID:AB_396514
**Chemicals, Peptides, and Recombinant Proteins**		
DPBS	Corning	Cat# 45000-434
Fatty Acid-Free BSA	Proliant Biologicals	Cat# 68700
D-(+)-Glucose Solution, 45%	Sigma-Aldrich	Cat# G8769
Accutase®	eBioscience	Cat# 00-4555-56
Penicillin-Streptomycin	Thermo Fisher Scientific	Cat# 15140122
GlutaMAX™	Thermo Fisher Scientific	Cat# 35050061
MEM Non-Essential Amino Acids Solution (100X)	Thermo Fisher Scientific	Cat# 11140050
Sodium Bicarbonate	Sigma-Aldrich	Cat# NC0564699
Matrigel®	Corning	Cat# 356231
ROCK Inhibitor Y-27632	STEMCELL Technologies	Cat# 72305
mTeSR1 Complete Kit - GMP	STEMCELL Technologies	Cat# 85850
RPMI 1640 1X, w/o L-Glutamine	Corning	Cat# 45000-404
DMEM/F12 with L-Glutamine, HEPES	Corning	Cat# 45000-350
DMEM/F12 w/o L-Glutamine	Corning	Cat# 45000-346
HyClone Dulbecco's Modified Eagles Medium	Thermo Fisher Scientific	Cat# SH30081.FS
MCDB 131	Thermo Fisher Scientific	Cat# 10372-019
CTS™ KnockOut™ SR XenoFree Kit	Thermo Fisher Scientific	Cat# A1099202
Insulin-Transferrin-Selenium (ITS-G) (100X)	Thermo Fisher Scientific	Cat# 41400045
Insulin-Transferrin-Selenium-Ethanolamine (ITS-X) (100X)	Thermo Fisher Scientific	Cat# 51500-056
B-27™ Supplement (50X)	Thermo Fisher Scientific	Cat# 17504044

Bovine Albumin Fraction V (7.5%)	Thermo Fisher Scientific	Cat# 15260037
2-Mercaptoethanol	Thermo Fisher Scientific	Cat# 21985-023
Human AB Serum	Valley Biomedical	Cat# HP1022
Activin A	R&D Systems	Cat# 338-AC/CF
Heregulinβ-1	Peprotech	Cat# 100-03
ALK5 Inhibitor II	Enzo Life Sciences	Cat# ALX-270-445
KGF/FGF7	R&D Systems	Cat# 251-KG
EGF	R&D Systems	Cat# 236-EG
Retinoic Acid	Sigma-Aldrich	Cat# R2625
LDN-193189	Stemgent	Cat# 04-0074
SANT-1	Sigma-Aldrich	Cat# S4572
TPB	Calbiochem	Cat# 565740
Noggin	R&D Systems	Cat# 3344-NG-050
Wnt3a	R&D Systems	Cat# 1324-WN-010
3,3',5-Triiodo-L-thyronine sodium salt (T3)	Sigma-Aldrich	Cat# T6397
TGFβ R1 kinase inhibitor IV	EMD Biosciences	Cat# 616454
KAAD-Cyclopamine	Toronto Research Chemicals	Cat# K171000
Heparin	Sigma-Aldrich	Cat# H3149
TTNPB	Sigma-Aldrich	Cat# T3757
Zinc Sulfate	Sigma-Aldrich	Cat# Z0251
TRIzol®	Thermo Fisher Scientific	Cat# 15596018
Polyethylenimine (PEI)	Polysciences	Cat# 23966-1
O.C.T. Compound	Sakura Finetek USA	Cat# 25608-930
Vectashield Antifade Mounting Medium	Vector Laboratories	Cat# H-1000
**Critical Commercial Assays**		
MinElute Reaction Cleanup Kit	QIAGEN	Cat#28204
miRNeasy Mini Kit	QIAGEN	Cat# 74104
mirVana™ miRNA Isolation Kit	Thermo Fisher Scientific	Cat# AM1560
Nextera DNA Library Preparation Kit (24 samples)	Illumina	Cat#FC-121-1030
TruSeq Stranded mRNA Library Preparation Kit	Illumina	Cat# 20020594
TruSeq Small RNA Library Preparation Kit	Illumina	Cat# RS-200-0012
RNA 6000 Nano Kit	Agilent Technologies	Cat# 5067-1511
High Sensitivity D1000 ScreenTape	Agilent Technologies	Cat# 5067-5584
RNA ScreenTape	Agilent Technologies	Cat# 5067-5576
RNA ScreenTape Sample Buffer	Agilent Technologies	Cat# 5067-5577
RNA ScreenTape Ladder	Agilent Technologies	Cat# 5067- 5578
Qubit ssDNA assay kit	Thermo Fisher Scientific	Cat# Q10212
iScript™ cDNA Synthesis Kit	Bio-Rad	Cat# 1708890
iQ™ SYBR® Green Supermix	Bio-Rad	Cat# 1708880
Cytofix/Cytoperm W/Golgi Stop Kit	BD Biosciences	Cat# 554715

**Deposited Data**		
RNA-seq and ATAC-seq data	GEO	GSE115327
**Experimental Models: Cell Lines**		
H1	WiCell Research Institute	NIHhESC-10-0043, RRID:CVCL_9771
CyT49	ViaCyte, Inc.	NIHhESC-10-0041, RRID:CVCL_B850
HEK293T	ATCC	Cat# CRL-3216, RRID:CVCL_0063
**Oligonucleotides**		
Assay ID: 000377, Taqman miRNA assay; hsa-let-7a	Thermo Fisher Scientific	Cat# 4427975
Assay ID: 002282, Taqman miRNA assay; hsa-let-7g	Thermo Fisher Scientific	Cat# 4427975
Assay ID: 000452, Taqman miRNA assay; hsa-mIR127- 3p	Thermo Fisher Scientific	Cat# 4427975
Assay ID: 000502, Taqman miRNA assay; hsa-mIR- 200a-3p	Thermo Fisher Scientific	Cat# 4427975
Assay ID: 002300, Taqman miRNA assay; hsa-mIR- 200c-3p	Thermo Fisher Scientific	Cat# 4427975
Assay ID: 000377, Taqman miRNA assay; hsa-let-7a	Thermo Fisher Scientific	Cat# 4427975
Assay ID: 000564, Taqman miRNA assay; hsa-mIR-375	Thermo Fisher Scientific	Cat# 4427975
Assay ID: 000268, Taqman miRNA assay; hsa-mIR-7- 5p	Thermo Fisher Scientific	Cat# 4427975
Assay ID: 000436, Taqman miRNA assay; hsa-mIR- 99b-5p	Thermo Fisher Scientific	Cat# 4427975
Assay ID: 001182, Taqman miRNA assay; hsa-mIR- 124-3p	Thermo Fisher Scientific	Cat# 4427975
Assay ID: 001094, Taqman miRNA assay; RNU control	Thermo Fisher Scientific	Cat# 4427975

ATTTgaattctcagccgcagatgcgttcaggtgagggcggaggctagcg<br>gggcgctgtgcagcactgagctcgcggaagaccaggaccaggagatca<br>c<br>cgagggcgaccgccaggccccgggccctccgctcccgccccgcgacga<br>gcccctcgcacaaaccggacctgagcgttttgttcgttcggctcgcgtgagg<br>caggggcggcctctcagcaccagcccgggggccggcctgatcgccacgc<br>aggcacctgccgccgccaccgccaccgccatctcaaccgtacgggtggg<br>agaggctgtgcgccgctccaggggagatccggctcccatccggccccacc<br>cgccctgccttgccctgcccgcagcttctTTCTTATCACTCACACA<br>GGAAACCAGGATTACCGAGGAGGAAAAAAAGCCTT<br>CCTGTGGTGCTCAACTGTGATTCCTTTTCACCATTC<br>ACCCTGGATGTTCTCTTCACTGTGGGATGAGgtAGT<br>A<br>GGTTGTATAGTTctgttgaatctcatggACTATACAATCTAC<br>TGTCTTTCCTAACGTGATAGAAAAGTCTGCATCCAG<br>GCGGTCTGATAGAAAGTCAGTTAACTAATTGTACAA<br>TATTTAAGATTAACTTGTCTTAAAGAGATGTAGTGC<br>A<br>GCATTTGTTTATGGCCTGGAAATAAATTAATTTAGA<br>G<br>ATAAAGTCTGTAGCAAGTACACTGGATGGGctccaaat<br>gtggtgcaagatgaggcaaatgtgtggcacttgtagctttgctgccaagcctc<br>tgctgtgaggatgttccctttcctgtctcaagtgcatcctgaagagttcctccag<br>cgctccgtttccttttgcctgattccaggctgaggtagtagtttgtacagttctgtt<br>gaatctcatggctgtacaggccactgccttgccaggaacagcgcgccagct<br>gccaagtggggctgagaggatggcgtcaccctgctcatctctgggaaacc<br>a<br>ggtaatggggaggaagtcCACCACCCCTGGCTGCTCACCG<br>CTCCGGTTCTTCCCTGGGCTTCCACAGCAGCCCCT<br>GCCTGCCTGGCGGGACCCCACGTCCCTCCCGGGC<br>CCCTGTGAGCATCTTACCGGACAGTGCTGGATTTC<br>C<br>CAGCTTGACTCTAACACTGTCTGGTAACGATGTTCA<br>AAGGTGACCCGCCGCTCGCCGGGGACACCACCGA<br>GGCACATCCGGAGCTCCTACTCCAGGGATGGGCTG<br>TTTTTTttaattaaGGTG	This study	IDT gBlock
Software and Algorithms		
Flowjo-v10	FlowJo LLC	http://www.flowjo.com/download-newest-version/
STAR 2.4.0f1	(Dobin et al., 2013)	https://github.com/alexdobin/STAR
Bowtie 1.1.1	(Langmead et al., 2009)	http://bowtie-bio.sourceforge.net/index.shtml
Cufflinks 2.2.1	(Trapnell et al., 2010)	https://github.com/cole-trapnell-lab/cufflinks
HTSeq 0.6.1	(Anders et al., 2015)	https://htseq.readthedocs.io/en/master/install.html
DEseq2 1.10.1	(Love et al., 2014)	https://www.bioconductor.org/packages/devel/bioc/html/DESeq2.html
MACS2	(Zhang et al., 2008)	http://liulab.dfci.harvard.edu/MACS/Download.html

BEDTools 2.17.0	(Quinlan and Hall, 2010)	https://bedtools.readthedocs.io/en/latest/content/installation.html
ComBat (part of the sva Bioconductor package)	(Johnson et al., 2007) (Leek, 2014)	http://bioconductor.org/packages/release/bioc/html/sva.html
GSEA	(Mootha et al., 2003; Subramanian et al., 2005)	http://www.broad.mit.edu/gsea
Metascape	(Tripathi et al., 2015)	http://metascape.org/gp/index.html#/main/step1
TargetScan	(Agarwal et al., 2015)	http://www.targetscan.org/vert_72/
JASPAR	(Khan et al., 2018)	http://jaspar.genereg.net/cgi-bin/jaspar_db.pl
Hocomoco	(Kulakovskiy et al., 2018)	http://hocomoco11.autosome.ru/
FIMO	(Grant et al., 2011)	http://meme-suite.org/tools/fimo
R 3.5.0		https://cran.r-project.org/
SAMtools 1.3	(Li et al., 2009)	https://github.com/samtools/samtools
HOMER 4.10	(Heinz et al., 2010)	http://homer.ucsd.edu/homer/download.html
HALO™ Image Analysis Software	PerkinElmer	http://www.perkinelmer.com/product/halo-plus-3-ws-license-cls141255
Adobe Illustrator CS5		
Adobe Photoshop CS5		

Supplemental References

Agarwal, V., Bell, G.W., Nam, J.W., and Bartel, D.P. (2015). Predicting effective microRNA target sites in mammalian mRNAs. Elife *4*.

Anders, S., Pyl, P.T., and Huber, W. (2015). HTSeq--a Python framework to work with high- throughput sequencing data. Bioinformatics 31, 166-169.

Aylward, A., Chiou, J., Okino, M., Kadakai, N., and Gaulton, K.J. (2018). Shared genetic contribution to type 1 and type 2 diabetes risk. bioRxiv.

Buenrostro, J.D., Giresi, P.G., Zaba, L.C., Chang, H.Y., and Greenleaf, W.J. (2013). Transposition of native chromatin for fast and sensitive epigenomic profiling of open chromatin, DNA-binding proteins and nucleosome position. Nature methods *10*, 1213-1218.

Chen, C.Z., Li, L., Lodish, H.F., and Bartel, D.P. (2004). MicroRNAs modulate hematopoietic lineage differentiation. Science *303*, 83-86.

Dobin, A., Davis, C.A., Schlesinger, F., Drenkow, J., Zaleski, C., Jha, S., Batut, P., Chaisson, M., and Gingeras, T.R. (2013). STAR: ultrafast universal RNA-seq aligner. Bioinformatics *29*, 15-21.

Gosline, S.J., Gurtan, A.M., JnBaptiste, C.K., Bosson, A., Milani, P., Dalin, S., Matthews, B.J., Yap, Y.S., Sharp, P.A., and Fraenkel, E. (2016). Elucidating MicroRNA Regulatory Networks Using Transcriptional, Post-transcriptional, and Histone Modification Measurements. Cell Rep *14*, 310-319.

Grant, C.E., Bailey, T.L., and Noble, W.S. (2011). FIMO: scanning for occurrences of a given motif. Bioinformatics 27, 1017-1018.

Heinz, S., Benner, C., Spann, N., Bertolino, E., Lin, Y.C., Laslo, P., Cheng, J.X., Murre, C., Singh, H., and Glass, C.K. (2010). Simple combinations of lineage-determining transcription factors prime cis-regulatory elements required for macrophage and B cell identities. Molecular cell *38*, 576-589.

Johnson, W.E., Li, C., and Rabinovic, A. (2007). Adjusting batch effects in microarray expression data using empirical Bayes methods. Biostatistics *8*, 118-127.

Kameswaran, V., Bramswig, N.C., McKenna, L.B., Penn, M., Schug, J., Hand, N.J., Chen, Y., Choi, I., Vourekas, A., Won, K.J.*, et al.* (2014). Epigenetic regulation of the DLK1-MEG3 microRNA cluster in human type 2 diabetic islets. Cell metabolism *19*, 135-145.

Khan, A., Fornes, O., Stigliani, A., Gheorghe, M., Castro-Mondragon, J.A., van der Lee, R., Bessy, A., Cheneby, J., Kulkarni, S.R., Tan, G., et al. (2018). JASPAR 2018: update of the open-access database of transcription factor binding profiles and its web framework. Nucleic Acids Res 46, D260-D266.

Kent, W.J., Sugnet, C.W., Furey, T.S., Roskin, K.M., Pringle, T.H., Zahler, A.M., and Haussler,
D. (2002). The human genome browser at UCSC. Genome Res *12*, 996-1006.

Kulakovskiy, I.V., Vorontsov, I.E., Yevshin, I.S., Sharipov, R.N., Fedorova, A.D., Rumynskiy,
E.I., Medvedeva, Y.A., Magana-Mora, A., Bajic, V.B., Papatsenko, D.A., et al. (2018). HOCOMOCO: towards a complete collection of transcription factor binding models for human and mouse via large-scale ChIP-Seq analysis. Nucleic Acids Res 46, D252- D259.

Langmead, B., Trapnell, C., Pop, M., and Salzberg, S.L. (2009). Ultrafast and memory-efficient alignment of short DNA sequences to the human genome. Genome Biol *10*, R25.

Leek, J.T. (2014). svaseq: removing batch effects and other unwanted noise from sequencing data. Nucleic Acids Res 42.

Li, H., and Durbin, R. (2009). Fast and accurate short read alignment with Burrows-Wheeler transform. Bioinformatics *25*, 1754-1760.

Li, H., Handsaker, B., Wysoker, A., Fennell, T., Ruan, J., Homer, N., Marth, G., Abecasis, G., Durbin, R., and Genome Project Data Processing, S. (2009). The Sequence Alignment/Map format and SAMtools. Bioinformatics *25*, 2078-2079.

Love, M.I., Huber, W., and Anders, S. (2014). Moderated estimation of fold change and dispersion for RNA-seq data with DESeq2. Genome Biol 15, 550.

Mootha, V.K., Lindgren, C.M., Eriksson, K.F., Subramanian, A., Sihag, S., Lehar, J., Puigserver, P., Carlsson, E., Ridderstrale, M., Laurila, E., et al. (2003). PGC-1alpha-responsive genes involved in oxidative phosphorylation are coordinately downregulated in human diabetes. Nature genetics 34, 267-273.

Piskounova, E., Viswanathan, S.R., Janas, M., LaPierre, R.J., Daley, G.Q., Sliz, P., and Gregory, R.I. (2008). Determinants of microRNA processing inhibition by the developmentally regulated RNA-binding protein Lin28. J Biol Chem *283*, 21310-21314.

Quinlan, A.R., and Hall, I.M. (2010). BEDTools: a flexible suite of utilities for comparing genomic features. Bioinformatics *26*, 841-842.

Rezania, A., Bruin, J.E., Arora, P., Rubin, A., Batushansky, I., Asadi, A., O'Dwyer, S., Quiskamp, N., Mojibian, M., Albrecht, T.*, et al.* (2014). Reversal of diabetes with insulin- producing cells derived in vitro from human pluripotent stem cells. Nat Biotechnol *32*, 1121-1133.

Subramanian, A., Tamayo, P., Mootha, V.K., Mukherjee, S., Ebert, B.L., Gillette, M.A., Paulovich, A., Pomeroy, S.L., Golub, T.R., Lander, E.S.*, et al.* (2005). Gene set enrichment analysis: a knowledge-based approach for interpreting genome-wide expression profiles. Proc Natl Acad Sci U S A *102*, 15545-15550.

Szklarczyk, D., Franceschini, A., Wyder, S., Forslund, K., Heller, D., Huerta-Cepas, J., Simonovic, M., Roth, A., Santos, A., Tsafou, K.P.*, et al.* (2015). STRING v10: protein- protein interaction networks, integrated over the tree of life. Nucleic Acids Res *43*, D447- 452.

Trapnell, C., Williams, B.A., Pertea, G., Mortazavi, A., Kwan, G., van Baren, M.J., Salzberg, S.L., Wold, B.J., and Pachter, L. (2010). Transcript assembly and quantification by RNA- Seq reveals unannotated transcripts and isoform switching during cell differentiation. Nat Biotechnol 28, 511-515.

Tripathi, S., Pohl, M.O., Zhou, Y., Rodriguez-Frandsen, A., Wang, G., Stein, D.A., Moulton, H.M., DeJesus, P., Che, J., Mulder, L.C.*, et al.* (2015). Meta- and Orthogonal Integration of Influenza "OMICs" Data Defines a Role for UBR4 in Virus Budding. Cell Host Microbe *18*, 723-735.

Xie, R., Everett, L.J., Lim, H.W., Patel, N.A., Schug, J., Kroon, E., Kelly, O.G., Wang, A., D'Amour, K.A., Robins, A.J.*, et al.* (2013). Dynamic chromatin remodeling mediated by polycomb proteins orchestrates pancreatic differentiation of human embryonic stem cells. Cell Stem Cell *12*, 224-237.

Zhang, H.M., Liu, T., Liu, C.J., Song, S., Zhang, X., Liu, W., Jia, H., Xue, Y., and Guo, A.Y. (2015). AnimalTFDB 2.0: a resource for expression, prediction and functional study of animal transcription factors. Nucleic Acids Res *43*, D76-81.

Zhang, Y., Liu, T., Meyer, C.A., Eeckhoute, J., Johnson, D.S., Bernstein, B.E., Nusbaum, C., Myers, R.M., Brown, M., Li, W.*, et al.* (2008). Model-based analysis of ChIP-Seq (MACS). Genome Biol *9*, R137.
